# Free Radical‐Mediated Photocyclization of Triphenylphosphindole Oxides for Photoactivated and Self‐Reported Lipid Peroxidation

**DOI:** 10.1002/advs.202305516

**Published:** 2023-10-23

**Authors:** Jianqing Li, Zeyan Zhuang, Jingjing Guo, Xiaobin Dong, Junyi Gong, Ben Zhong Tang, Zujin Zhao

**Affiliations:** ^1^ State Key Laboratory of Luminescent Materials and Devices Key Laboratory of Luminescence from Molecular Aggregates of Guangdong Province South China University of Technology Guangzhou 510640 China; ^2^ School of Chemistry Chemical Engineering and Biotechnology Nanyang Technological University 21 Nanyang Link Singapore 637371 Singapore; ^3^ School of Science and Engineering Shenzhen Institute of Aggregate Science and Technology The Chinese University of Hong Kong Shenzhen Guangdong 518172 China

**Keywords:** aggregation‐induced emission, lipid peroxidation, phosphindole oxide, photocyclization, photodynamic therapy

## Abstract

Photocyclization is demonstrated as a powerful tool for building complicated polycyclic molecules. And efficient photocyclization is competent as an artful strategy to develop photo‐responsive smart materials. Herein, an efficient free radical‐mediated photocyclization for triphenylphosphindole oxide (TPPIO) derivatives to generate tribenzophosphindole oxide (TBPIO) derivatives at ambient condition is reported. The reaction mechanism and substituent effect on photocyclization efficiency are thoroughly investigated. Additionally, photophysical and photochemical properties of TPPIO and TBPIO derivatives are measured for comparison and deeply deciphered by theoretical calculation. TPPIO derivatives own typical aggregation‐induced emission feature but barely generate reactive oxygen species (ROS), while TBPIO derivatives experience aggregation‐caused quenching but show efficient Type I ROS generation capacity. Further, in vitro experiments demonstrate that this photo‐conversion can efficiently occur in situ in living cells to activate photodynamic therapy (PDT) effect to trigger lipid peroxidation with selective fluorescence “light up” in lipid droplet area under continuous irradiation. This work extends the optoelectronically and biologically interesting phosphindole oxide‐containing π‐conjugated systems through an efficient synthetic strategy, provides in‐depth mechanistic descriptions in the aspects of reaction and property, and further presents their great potentials for photoactivated and self‐reported PDT.

## Graphical abstract

An efficient free radical‐mediated photocyclization for triphenylphosphindole oxide derivatives to generate tribenzophosphindole oxide derivative is reported. Interestingly, this photo‐conversion can efficiently occur in living cells to activate photodynamic therapy and trigger fluorescence “light up” in lipid droplet area under continuous irradiation.

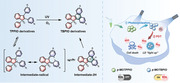

## 1 Introduction

Photochemistry has been developed as a powerful tool for organic synthesis, allowing to access structurally elaborate organic molecules from simple building blocks.^[^ ^1^ ^]^ Photochemical syntheses have many merits of excellent regioselectivity, high yield, mild condition, and environmental protection.^[^ ^2^ ^]^ Generally, photochemical reactions are initiated when organic molecules harvest photons with specific wavelengths to reach excited states with fundamentally different electronic configurations from those in ground state, which change the chemical activity of the molecules and broaden the reaction diversity relative to the thermal reactions.^[^ ^3^ ^]^ Among various categories of photochemical reactions, photo‐induced [6π] electrocyclization plays an important role in the efficient construction of functionalized polycyclic skeletons. Over the past few years, [6π] photocyclization has been utilized to build a great number of intricate natural products (e.g., dictyodendrins B, methoxatin, ellipticine, staurosporine aglycon)^[^ ^4^ ^]^ and artificial materials (e.g., photochromic switches, super‐resolution imaging agents, chemosensor).^[^ ^5^ ^]^ In spite of these significant advances, the potential of [6π] photocyclization in developing functional scaffolds with structural diversity has not been fully untapped and the mechanisms involved still require further investigation.^[^ ^6^ ^]^ Expanding the toolset of [6π] photocyclization will continue to drive forward the application of photochemistry in not only organic synthesis methodology but also advanced functional materials.

By exploiting the photoreaction‐induced changes in molecular properties, efficient photochemical transformations have huge potential in building photo‐responsive smart materials, and have been widely explored in the areas of data processing, information security, health care, soft robotics, and so on.^[^ ^7^ ^]^ Particularly in biological field, photo‐responsive materials with unique advantages of precise and remote control, environmental friendliness and non‐invasiveness have opened a door for the advanced technologies, such as super‐resolution imaging, photo‐controlled drug release, photo‐activated photodynamic therapy (PDT), and biomimetic artificial muscles. Nevertheless, there is still plenty of room for further expansion and improvement of photo‐responsive biomaterials, even based on the existing photochemical methodology.^[^ ^8^ ^]^ In response, designing new photochemical reaction involved reactants and products with markedly different properties can offer a great opportunity to develop novel photo‐responsive biomaterials with desired performance.

**Scheme 1:**
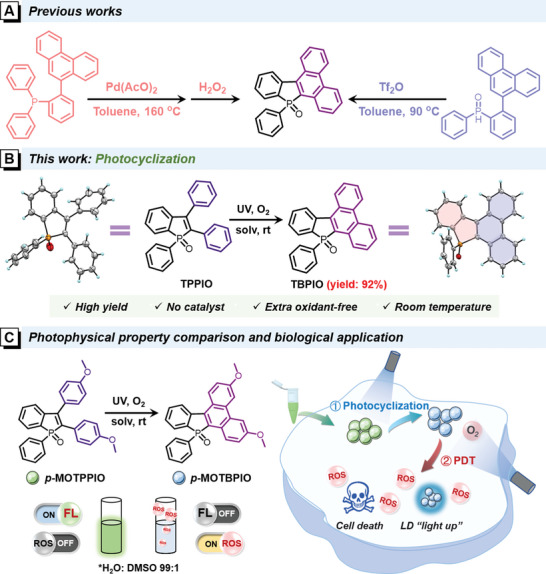
A) Reported synthetic routes of TBPIO. B) Synthetic route of TBPIO by photocyclization in this work. C) Synthetic route of *p*‐MOTBPIO from *p*‐MOTPPIO by photocyclization and the schematic diagram of their photophysical property comparison and biological application exploration (solv: solution; rt: room temperature; FL: fluorescence; PDT: photodynamic therapy; ROS: reactive oxygen species; LD: lipid droplet)

Phosphine oxide (P≐O) based π‐conjugated systems have attracted considerable attention as useful building units for functional organic materials in the fields of optoelectronic devices,^[^ ^9^ ^]^ chemical sensors, and disease diagnosis and therapy,^[^ ^10^ ^]^ etc., because of their intriguing electronic structures and geometric configurations.^[^ ^11^ ^]^ The pentavalent phosphoryl is an electron‐withdrawing group with low reduction potential, corresponding to low‐lying lowest unoccupied molecular orbital (LUMO) level and high stability, due to the electronegativity of oxygen and σ*−π* orbital hyperconjugation. In addition, the P(sp^3^) tetrahedron configuration of P≐O group capacitates the well‐tailored substituents to modulate molecular conformation and intermolecular interaction. Besides, the versatile reactivity of the P center allows further structural modification towards functionalized modules with tunable properties. Despite these superiorities, so far, the syntheses of P≐O‐containing polycyclic structures have been subject to laborious procedures with crucial drawbacks. For instance, as shown in **Scheme** **1**A, conventional synthetic methods of tribenzophosphindole oxide (TBPIO) require tedious sequences at high temperatures, expensive metal catalysts, aggressive reagents, and/or complicated precursors.^[^ ^12^ ^]^ In this context, photocyclization reactions mentioned above can provide a potentially viable approach for rapidly and easily obtaining P≐O‐containing polycyclic structures, like TBPIO and its derivatives, yet, which has rarely been reported.

So, in this work, we wish to report an efficient photocyclization reaction that can transform triphenylphosphindole oxide (TPPIO) to corresponding polycyclic TBPIO under UV irradiation at ambient condition (Scheme 1B). An in‐depth study is subsequently conducted on the reaction mechanism and substitute effect. In addition, the photophysical properties of the generated TBPIO derivatives are investigated and their application potentials in image‐guided and photo‐activated PDT are evaluated (Scheme 1C). An interesting reversal is observed that the TPPIO derivatives have typical aggregation‐induced emission (AIE) characteristics.^[^ ^13^ ^]^ But poor photodynamic reactive oxygen species (ROS) generation abilities, while the TBPIO derivatives exhibit aggregation‐caused quenching (ACQ) features with efficient photodynamic ROS generation capacities. Such kind of photo‐conversion can efficiently occur in situ in living cells to activate PDT with selective “light up” phenomena in lipid droplet (LD) under continuous irradiation.

## 2 Results and Discussion

### 2.1 Synthesis of TPPIO Derivatives

**Scheme 2:**
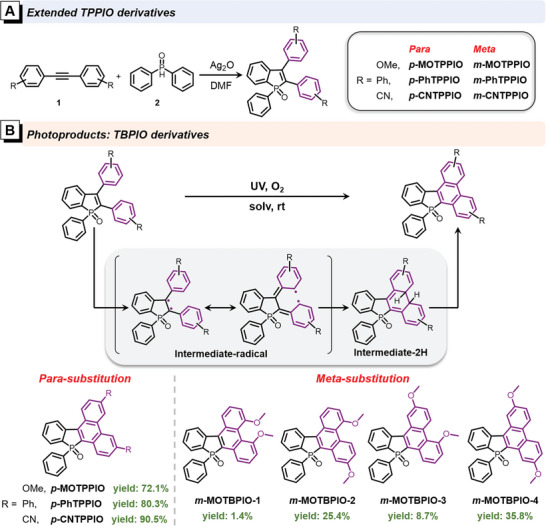
A) Synthetic routes of TPPIO derivatives. B) Synthetic routes and molecular structures of TBPIO derivatives, indicated with reaction yields (under N_2_ atmosphere with a small amount of dissolved oxygen), and proposed photocyclization mechanism.

As illustrated in **Scheme** **2**A, a series of TPPIO derivatives with diverse substituents of methoxyl (MeO), phenyl (Ph), and cyano (CN) at *para*‐ and *meta‐*positions are designed and synthesized. Intermediate **1** is smoothly prepared by Sonogashira coupling according to the literature method, followed by an oxidative cyclization with diphenylphosphine oxide (**2**) under the catalysis of silver oxide (Ag_2_O). TPPIO derivatives are obtained in high yields (over 85%), whose structures are characterized by nuclear magnetic resonance (^1^H and ^13^C NMR), high‐resolution mass spectroscopy (HRMS), and X‐ray diffraction (XRD) as shown in Supporting Information. It is noteworthy that this mature synthetic route allows for the introduction of a large variety of substituents by chemical modification on the starting diphenylethlene derivatives.

### 2.2 Photocyclization Reactions and Photoproducts

**Figure 1:**
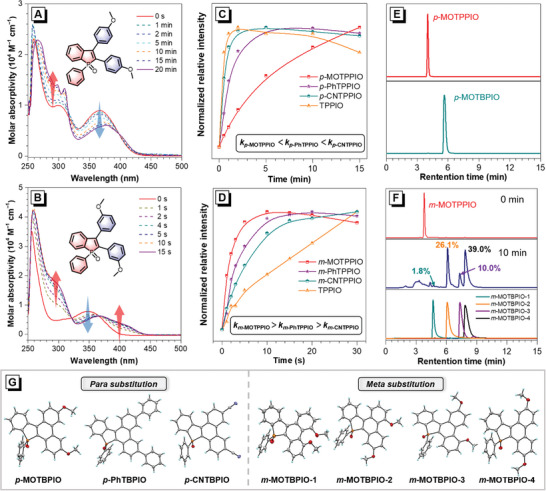
UV–vis absorption spectra of A) *p*‐MOTPPIO and B) *m*‐MOTPPIO in DMSO (10 µm) upon UV light irradiation for different time. Insets: Molecular structures. C,D) Normalized signal changes of absorption intensity at a fixed wavelength of TPPIO and its derivatives upon UV light irradiation (365 nm, 40 mW cm^−2^) for different time. (*p*‐MOTPPIO: 310 nm, *p*‐PhTPPIO: 310 nm, *p*‐CNTPPIO:300 nm, TPPIO: 390 nm, *m*‐MOTPPIO: 300 nm, *m*‐PhTPPIO: 310 nm, *m*‐CNTPPIO: 300 nm). HPLC spectra of E) *p*‐MOTPPIO and F) *m*‐MOTPPIO before and after UV light irradiation (365 nm, 40 mW cm^−2^), and the cyclization products *p*‐MOTBPIO and *m*‐MOTBPIO. (G) Crystal structures of TBPIO derivatives.

These TPPIO derivatives hold interesting photochromic behaviors, and deserve detail investigation. For example, as shown in **Figure** **1**A, in dilute dimethylsulfoxide (DMSO) solution (10 µM), *p*‐MOTPPIO with MeO substituents at *para‐*positions shows greatly changed absorption spectral profiles with gradually decreased and red‐shifted absorption maximum, upon UV light irradiation (365 nm, 40 mW cm^−2^). A similar phenomenon is observed in *m*‐MOTPPIO, but with a much faster photochromic response relative to *p*‐MOTPPIO, and noticeable red‐shift and decrease in absorption maximum with a rising shoulder peak is recorded in absorption spectrum within a few seconds (Figure 1B). Then, the substituent effect on reaction kinetics is investigated by fully recording the photochromic behaviors of TPPIO and its derivatives (Figure S1, Supporting Information) and extracting the change of the absorption intensity at a fixed wavelength upon UV irradiation (Figure 1C,D). It is found that both substitution positions and electronic effects of the substituents pose an apparent influence on the photoreaction efficiency. Generally, the *meta‐*substituted TPPIO derivatives show the highest photochromic responsive efficiencies, bare TPPIO ranks second, and *para‐*substituted ones are the most sluggish, indicating the *meta‐*substitution is conducive to the occurrence of this reaction. But *para‐*substitution is vice versa. More interestingly, for the *para‐*substituted TPPIO derivatives, their photochromic response rates are found to increase along with the enhancement of electron‐withdrawing ability of the substitutes (*p*‐MOTPPIO < *p*‐PhTPPIO < *p*‐CNTPPIO), while for the *meta‐*substituted ones, an opposite trend is recorded (*m*‐MOTPPIO > *m*‐PhTPPIO > *m*‐CNTPPIO). These findings reveal that the electron‐withdrawing substituents are beneficial to the photocyclization of *para‐*substituted TPPIO derivatives, while the electron‐donating substituents are good for the *meta‐*substituted ones, reflecting the orientation effect of substituents on benzene rings.^[^ ^14^ ^]^

As follows, high‐performance liquid chromatography (HPLC) is employed to monitor the photoresponse process, taking *m*‐MOTPPIO as an example. As illustrated in Figures 1F, the peak of *m*‐MOTPPIO is disappeared and more than one newly appeared peaks are recorded after irradiation for 20 min under air (1 mg mL^−1^, acetonitrile solution), signifying that various phtotproducts are yielded. Notably, under strict oxygen‐free conditions (the flask containing reaction solution is frozen with liquid nitrogen and then subjucted to three vacuum extraction and N_2_ filling), the photoreaction is effectively inhibited. As shown in Figure S2 (Supporting Information), the new peaks from photoproducts are negligible after 20 min irradiation, indicating the participation of O_2_ is necessary in this photoreaction. To acquire the main products from the photoreaction for further investigation, hundred milligram‐scale photoreactions of these TPPIO derivatives are performed in methanol (≈10 mg mL^−1^) at room temperature. Delightfully, it is found that photoreaction can also take place under microoxygen environment. The reaction rates and yields under air and N_2_ atmosphere (the reaction flask was vacuumed and N_2_ filled for three times at room temperature) upon UV light irradiation (365 nm, 40 mW cm^−2^) are quantitatively compare. Thin layer chromatography is employed to monitor the reaction process. It is found that the photoreaction can be run under N_2_ atmosphere with close reaction rates but less by‐products, relative to air atmosphere, probably due to that the trace amount of dissolved oxygen in solvent is exactly ample to participate in the photoreaction but insufficient to promote the side reactions.^[^ ^15^ ^]^ The reaction rates of these TPPIO derivatives are basically consistent with their photochromic response rates. It takes ≈6 h for the *meta‐*substituted TPPIO derivatives to complete the reaction, and 12−16 h for TPPIO and *p*‐CNTPPIO. And for *p*‐MOTPPIO and *p*‐PhTPPIO, a small amount of reactants remain after 72 h. It should be noted that, with the prolongation of the irradiation time, more and more undesired by‐products generate in reaction mixtures, especially under air atmosphere.

After the reaction, the crude photoproducts are extracted by silica‐gel column chromatography, and then characterized and analyzed by NMR and HPLC. It is found that TPPIO and its *para‐*substituted derivatives yield sole photocyclization products (Figure 1E and Figure S3, Supporting Information). These products can be easily obtained by further recrystallization in good yields of 72−92% under N_2_ atmosphere and relatively lower yields under air atmosphere (33−88%) (Table S1, Supporting Information). The structures of TBPIO, *p*‐CNTBPIO, *p*‐MOTBPIO, and *p*‐PhTBPIO are further verified by single‐crystal XRD (Figure 1G). However, all the crude photoproducts from the *meta*‐substituted TPPIO derivatives are found to have isomers. And more than one newly appeared peaks are also recorded in HPLC spectra (Figure S4, Supporting Information), signifying that a mixture of several isomers with different cyclization sites are yielded, as the case of *m*‐MOTPPIO shown in Scheme 2B. Then, *semi‐*preparative HPLC is employed to separate the isomer mixtures, and four photoproducts of *m*‐MOTPPIO are successfully isolated. According to the retention time in HPLC curve from short to long, four *m*‐MOTBPIO isomers are named from *m*‐MOTBPIO‐1, *m*‐MOTBPIO‐2, *m*‐MOTBPIO‐3, and *m*‐MOTBPIO‐4 with yields of 1.4%, 25.4%, 8.7%, and 35.8%, respectively, under N_2_ atmosphere. Single crystals of these four photoproducts are successfully obtained, thus offering solid evidence to confirm the specific structures of these isomers (Figure 1G). Their detailed NMR, HRMS, and crystal data are given in Supporting Information. Among four isomers, the yield of *m*‐MOTBPIO‐4 with two cyclization sites at the *para*‐positions of the substituents is the highest and that of *m*‐MOTBPIO‐1 with two cyclization sites at the *ortho*‐positions is the lowest, mainly due to the steric effect from the substituents.

In addition, to test whether this photoreaction can occur in solid state, the powders of *p*‐MOTPPIO and *m*‐MOTPPIO are irradiated by UV light (365 nm, 40 mW cm^−2^) for 12 h. However, neither the color of powders nor the ^1^H NMR spectra show apparent change (Figure S5, Supporting Information), indicating that this photocyclization reaction is highly suppressed in the solid state, which should be ascribed to that the severe spatial constraint in condensed phase restrict the molecules to adjust their twisted conformations to form planar ones for the occurrence of the reaction.

### 2.3 Mechanism of Photocyclization Reactions

According to previous work,^[^ ^6b^ ^]^ it is presupposed that this photocyclization reaction firstly involves an intermediate with biradicals generated from the double‐bonded π‐electron of antiaromatic phosphene and resonating along two phenyl rings attached to the phosphene, followed by bonding and dehydrogenation to yield TBPIO derivatives (Scheme 2B). To gain in‐depth insights into the photocyclization mechanism, both experimental and theoretical investigations are carried out as follows.

**Figure 2:**
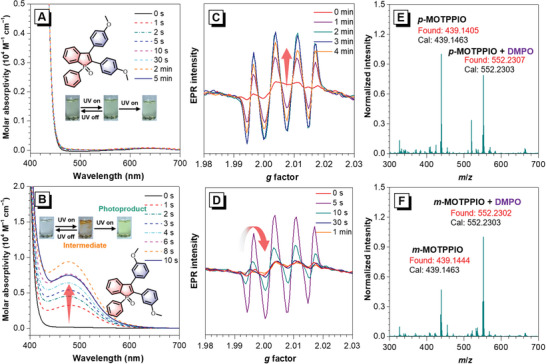
UV–vis absorption spectra of A) *p*‐MOTPPIO and B) *m*‐MOTPPIO in DMSO (5 mm) upon UV light irradiation for different time. Insets: molecular structures and photographs (shot under daylight) of color changes upon UV light irradiation (365 nm, 40 mW cm^−2^). EPR spectra of C) *p*‐MOTPPIO and D) *m*‐MOTPPIO in the presence of DMPO (25 mm) in DMSO (1 mm) upon UV light irradiation for different time (365 nm, 40 mW cm^−2^). HRMS spectrum of photo‐irradiated E) *p*‐MOTPPIO and F) *m*‐MOTPPIO in THF with the addition of 8 equiv.

The photochromism behaviors of TPPIO derivatives at high concentrations (5 mm in dichloromethane) are first studied in an attempt to capture the signals from the intermediates. Although no significant variations in the range above 400 nm are observed for all the *para‐*substituted TPPIO derivatives (**Figure** **2**A; Figure S6B, 6C, Supporting Information) and and *m*‐CNTPPIO (Figure S6E, Supporting Information) upon UV light irradiation (365 nm, 40 mW cm^−2^). TPPIO (Figure S6A, Supporting Information), *m*‐MOTPPIO (Figure 2B), and *m*‐PhTPPIO (Figure S6D, Supporting Information) show similar changes with evidently increased absorption bands at long‐wavelength region. Taking *m*‐MOTPPIO as an example, a visible color alteration from colorless to orange is vivid along with an emerging peak centered at about 475 nm, which tends to increase with the continuous irradiation, but spontaneously restores and decolorizes within a few seconds if the irradiation is removed timely. After continuous irradiation for about 10 s, the new absorption band gradually decreases and disappears, and the solution irreversibly converts from orange to pale yellow, implying the generation of unstable intermediates. This emerging absorption band at long‐wavelength region could be associated with the cyclized undehydrogenated intermediate with a slightly distorted π‐conjugated molecular plane, as the examples of *m*‐MOTPPIO‐2H shown in Scheme 2B.^[^ ^16^ ^]^ These findings suggest the efficient radical generation and bonding, but relatively inefficient dehydrogenation of TPPIO, *m*‐MOTPPIO, and *m*‐PhTPPIO, so that the long‐lived cyclized undehydrogenated intermediates can be observed. It is proposed that the weaker reactivity of the *para‐*substituted TPPIO derivatives limits the formation of the biradical intermediates, while the additional cyano groups of *m*‐CNTPPIO may promote dehydrogenation. Thus, no absorption signals are found for these molecules.

To verify the participation of radical intermediates, electron paramagnetic resonance (EPR) measurement is employed by using 5,5‐dimethyl‐1‐pyrroline‐1‐oxide (DMPO) as spin‐trapping reagent.^[^ ^17^ ^]^ As depicted in Figure 2C,D and Figure S7 (Supporting Information), obvious quartet EPR signals with *g* factors of 1.99−2.02 are monitored in all TPPIO derivatives (1 mm in DMSO) after exposure to UV light irradiation (365 nm, 40 mW cm^−2^), indicative of the formation of radical species during the photocyclization process. Notably, the radical signals for the *meta‐*substituted TPPIO derivatives appear within a short‐time irradiation of 5 s, and vanish immediately, while for the *para‐*substituted ones, a much longer irradiation time of several minutes is required to get clear radical signals, which can persist for a longer time as well. These results demonstrate the better radical generation efficiency and higher radical activity of the *meta‐*substituted TPPIO derivatives, in good agreement with their photochromism behaviors. Moreover, HRMS is measured to verify the radical monoadducts of TPPIO derivatives and DMPO (Figures 2E,F and Figure S8, Supporting Information). For example, after UV light irradiation (365 nm, 40 mW cm^−2^), both *p*‐MOTPPIO and *m*‐MOTPPIO show new peaks at *m/z* 552.2307 and 552.2302, respectively, which correspond to the molecular mass of their DMPO monoadducts. As expected, there is no EPR signal detected for the powder under UV irradiation (Figure S9, Supporting Information), proving again the absence of the photoreaction in solid state.

**Figure 3:**
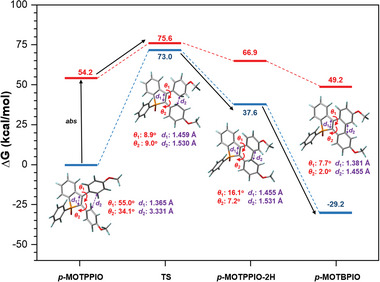
Relative energy profile for the photocyclization reaction coordinates of *p*‐MOTPPIO (blue lines: ground state; red line: first excited state; blank arrows: reaction path). Inset: optimized intermediate configurations with labelled dihedral angles (*Θ*_1_, *Θ*_2_) and C−C distances (*d*_1_, *d*_2_).

To deeply decipher the mechanism under this photocyclization reaction, a theoretical simulation of the case of *p*‐MOTPPIO is performed at the level of PBE0/def2‐SVP to identify reaction coordinates by nudged elastic band (NEB) method based on density functional theory (DFT) and time‐dependent density functional theory (TD‐DFT) calculations, using ORCA^[^ ^18^ ^]^ and Multiwfn.^[^ ^19^ ^]^ As displayed in **Figure** **3**, upon vertical excitation at the gound state, *p*‐MOTPPIO is stimulated to the first singlet excited (S_1_) state with high relative energy of 54.2 kcal mol^−1^. Then, an energy barrier of 21.4 kcal mol^−1^ in S_1_ state should be overcome from the ground state configuration to the transition state (TS)‐configuration, which is thermodynamically allowed at room temperature. Conversely, a large energy barrier of 73.0 kcal mol^−1^ in ground state accounts for why this photocyclization reaction cannot happen in the absence of light irradiation. Here, the TS configuration is found to own a prolonged C≐C bond in the phosphene ring (*d*_1_ = 1.459 Å) and a shorter distance between two phenyl rings (*d*_2_ = 1.530 Å) with smaller torsion angles between phosphene and two phenyl rings (*Θ*_1_ = 8.9^o^ and *Θ*_2_ = 9.0^o^), relative to the ground state configuration of *p*‐MOTPPIO (*d*_1_ = 1.365 Å, *d*_2_ = 3.331 Å, *Θ*_1_ = 55.0^o^ and *Θ*_2_ = 34.1^o^). Notably, a great deformation occurs in two phenyl rings, where two closest carbons tend to adopt a sp^3^ configuration with an unbond orbital that can accept a radical. These results indicate that, in the TS configuration, a broken π‐bond in the phosphene produce a pair of biradicals that can be transferred to two adjacent phenyl rings to form a resonance structure. Subsequently, this highly unstable biradical intermediate floats down from first excited state to ground state along the extremely small energy gap (2.6 kcal mol^−1^), followed by a relaxation into a cyclized undehydrogenated intermediate, *p*‐MOTPPIO‐2H. In *p*‐MOTPPIO‐2H, two deformed phenyl rings are slightly restored with an increased torsion angle of *Θ*_1_ (16.1^o^) and a single bond is formed between the two closest carbons of two phenyl rings.

It is noted that the transition energy of *p*‐MOTPPIO‐2H from ground state to S_1_ state is smaller than that of *p*‐MOTPPIO, due to the better conjugation of *p*‐MOTPPIO‐2H, which supports that a cyclized undehydrogenated intermediate could have a slightly red‐shifted absorption band relative to the precursor. Then, *p*‐MOTPPIO‐2H undergoes oxidative dehydrogenation in the presence of oxygen as an oxidizing agent, neatly yielding the final oxidative cyclization product, *p*‐MOTBPIO, carrying a more complanate polycyclic π‐conjugated structure. In addition, *p*‐MOTBPIO has lower Gibbs energy than *p*‐MOTPPIO in ground state, manifesting the formation of such kind of photocyclization reaction is thermodynamically preferred.

### 2.4 Photophysical Properties and ROS Generation

**Figure 4:**
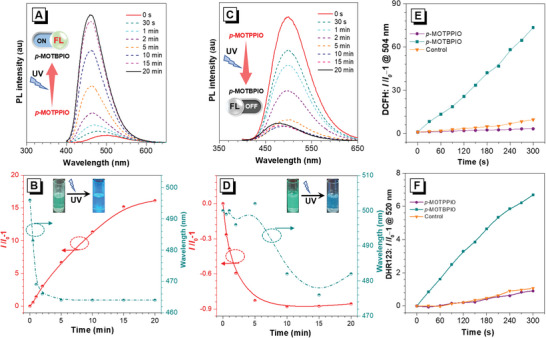
Fluorescence spectra of *p*‐MOTPPIO (10 µm) A) in DMSO and C) in mixtures of water and DMSO (*f*_w_ = 99 vol%) upon UV light irradiation for different time. Plots of *I*/*I*_0_ ‒ 1 and PL peak vs. irradiation time of *p*‐MOTPPIO (10 µm) B) in DMSO and D) in the mixtures of water and DMSO (*f*_w_ = 99 vol%), where *I*_0_ is the fluorescence intensity of *p*‐MOTPPIO before irradiation. Insets: Photographs (shot under 365 nm UV light) of fluorescence before and after UV light irradiation. E) General ROS and F) free radical ROS generation of *p*‐MOTPPIO (10 µm) and *p*‐MOTBPIO (10 µm) in water and DMSO (*f*_w_ = 99 vol%) using DCFH and DHR123 as indicators, respectively, under white light irradiation, where *I*_0_ is the fluorescence intensity of indicators before irradiation (UV light: 365 nm, 40 mW cm^−2^; white light: 10 mW cm^−2^).

Besides the absorption variation shown above, the differences in photo‐related properties between TPPIO and TBPIO derivatives are investigated to provide guidance for the further development of their application potentials. These TPPIO derivatives have typical molecular structures of a stator and several rotators for AIE luminogens.^[^ ^20^ ^]^ Hence, AIE characters are firstly evaluated by measuring the photoluminescence (PL) spectra in water/DMSO mixtures with varied water fractions (*f*_w_s), taking *p*‐PhTPPIO as an example. As shown in Figure S10A,B (Supporting Information), by increasing *f*_w_, *p*‐PhTPPIO shows gradually enhanced PL intensity at an unaltered peak of 500 nm. Meanwhile, the fluorescence quantum yields (*Ф*_F_s) of *p*‐PhTPPIO are recorded to be 12.4% in solution and 75.5% in film (Table S2, Supporting Information). And the *α*_AIE_ values (*Ф*_F_ (solid)/*Ф*_F_ (solution)) of these TPPIO derivatives are calculated to be ≈6−20. These results verify the AIE feature of the TPPIO derivatives. Inversely, for the photoproduct, *p*‐PhTBPIO has a blue‐shifted PL peak at 460 nm, and displays decreased PL intensity with the increase of *f*_w_ (Figure S10C,D, Supporting Information). And *p*‐PhTBPIO shows a higher *Ф*_F_ of 65.4% in solution but a lower *Ф*_F_ of 16.8% in solid, presenting distinct ACQ phenomenon. Similar ACQ characteristics are also observed in other TBPIO derivatives. The decreased *Ф*_F_s of TBPIO derivatives in solid should be ascribed to the planar and rigid TBPIO core, which can experience strong intermolecular interactions and thus non‐radiative decay.^[^ ^21^ ^]^ Taking the advantage of conversion from AIE to ACQ caused by this photocyclization reaction, opposite photo‐response behaviors are likely to be achieved (**Figure** **4**A−D). For *p*‐MOTPPIO, along with the elongation of irradiation time, it shows “turn on” fluorescence with peaks moving from 497 to 464 nm in good solvent of DMSO (Figure 4A,B, and Figure S11, Supporting Information), while it shows “turn off” fluorescence with peaks shifting from 500 to 480 nm in poor solvent (mixtures of water and DMSO with *f*_w_ = 99 vol%) (Figure 4C,D), well consistent to the AIE features of the precursors and the ACQ characters of the photoproducts.

According to previous reports, this kind of TPPIO analogues could behavior as photosensitizer (PS) for PDT.^[^ ^22^ ^]^ Then, the ROS generation capacities of the representative *p*‐MOTPPIO and *p*‐MOTBPIO in aqueous media are evaluated. The general ROS generation is firstly detected using 2,7‐dichlorodihydrofluorescein (DCFH) as indicator.^[^ ^23^ ^]^ As depicted in Figure 4E and Figure S12 (Supporting Information), the PL intensity from DCFH gradually increases and achieves ≈74‐fold enhancements after white light irradiation (10 mW cm^−2^) for 5 min in the presence of *p*‐MOTBPIO aggregates, while those in *p*‐MOTPPIO and control groups are barely enhanced under the same condition. According to the generation mechanisms, ROS can be divided into Type I (superoxide (O_2_^•‒^), peroxide (O_2_^2‒^), and hydroxyl (HO^•^)) ROS and Type II (singlet oxygen, ^1^O_2_) ROS.^[^ ^24^ ^]^ To evaluate the nature of ROS, dihydrorhodamine 123 (DHR 123) and 9,10‐anthracenediyl‐bis(methylene) dimalonic acid (ABDA) are utilized to distinguish the type of ROS.^[^ ^25^ ^]^ As shown in Figure 4F and Figure S13 (Supporting Information), the fluorescence of DHR123 enhances significantly in the presence of *p*‐MOTBPIO upon white light illumination (10 mW cm^−2^), whereas the fluorescence in the other groups keeps faint after irradiation. However, no significant absorption change is recorded for ABDA in the presence of *p*‐MOTPPIO or *p*‐MOTBPIO (Figures S14 and S15, Supporting Information) under the same condition. Moreover, hydroxyphenyl fluorescein (HPF), a HO^•^ indicator, is employed to further determine the type of free radial. However, no obvious fluorescence signals are detected for *p*‐MOTPPIO and *p*‐MOTBPIO, indicating the absence of HO^•^ (Figure S16, Supporting Information). These results indicate that *p*‐MOTBPIO holds excellent free radical ROS (except HO^•^) generation capacity, while *p*‐MOTPPIO owns almost no ROS generation capacity. In other words, the photoproducts have great potential as efficient PSs and the precursors are promising as photoactivated PSs for anticancer/antibacterial treatment.

In addition, differing from *p*‐MOTPPIO with high photo‐reactivity, the photostability of *p*‐MOTBPIO is verified satisfactory. As shown in Figure S17, Supporting Information, after 1 h continuous irradiation by 365 nm UV light, almost no changes are detected in both absorption and emission spectra of *p*‐MOTBPIO. And the inconspicuous PL spectral alteration of *p*‐MOTBPIO in solution with different polarity and viscosity indicates its limited sensitivity to polarity and viscosity (Figures S18 and S19, Supporting Information).

### 2.5 Mechanisms of Photophysical Properties and ROS Generation

**Figure 5:**
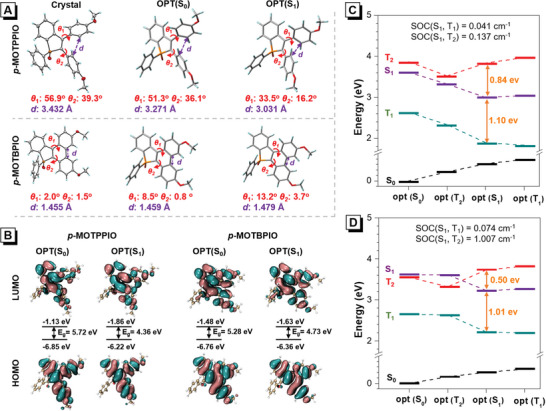
A) Crystal and optimized structures in S_0_ and S_1_ states with labelled dihedral angles (*Θ*_1_, *Θ*_2_) and the smallest C‒C distance (*d*) between two phenyl rings at the level of M06‐2X/6‐31G(d), using G16. B) HOMO and LUMO of *p*‐MOTPPIO and *p*‐MOTBPIO in the optimized S_0_ and S_1_ states. Calculated energy diagram at the optimized S_0_, S_1_, T_1_, and T_2_ states of C) *p*‐MOTPPIO and D) *p*‐MOTBPIO with labeled SOC values.

To understand the mechanism behind the photophysical behaviors of TPPIO derivatives and their photocyclization products, the conformations of *p*‐MOTPPIO and *p*‐MOTBPIO and other TPPIO derivatives in ground state (S_0_) and S_1_ state are optimized under the solvent field of DMSO, using G16 package (Figure S20 and Table S3, Supporting Information).^[^ ^26^ ^]^ As shown in **Figure** **5**A, *p*‐MOTPPIO owns a phosphindole stator and three phenyl rotors attached at the 1,2,3‐positions of phosphindole, while *p*‐MOTBPIO has a planar polycyclic tribenzophosphindole with a phenyl ring attached at the 1‐position. For both molecules, the highest occupied molecular orbits (HOMOs) and the lowest unoccupied molecular orbits (LUMOs) in S_0_ and S_1_ states are distributed on all the molecules except for the phenyl ring at the 1‐position, which dominate S_0_→S_1_ transition at S_0_ state and S_1_→S_0_ transition at S_1_ state, presenting apparent local excitation character (Figure 5B). In S_1_ state, *p*‐MOTPPIO shows a smaller HOMO‐LUMO energy gap (4.36 eV), relative to *p*‐MOTBPIO (4.75 eV), theoretically accounting for the bluer emission of *p*‐MOTBPIO. Meanwhile, the more rigid structure of *p*‐MOTBPIO relative to *p*‐MOTPPIO can reduce reorganization energy, which can contribute to bluer emission as well.^[^ ^27^ ^]^

To understand the ROS generation mechanism, the energy level diagrams of *p*‐MOTPPIO and *p*‐MOTBPIO at the different optimal states (S_0_, S_1_, T_1_, and T_2_) are illustrated in Figure 5D,E. Generally, small energy gaps between singlet and triplet states with considerable spin‐orbital coupling (SOC) constants are beneficial to intersystem crossing (ISC) and ROS generation.^[^ ^28^ ^]^ Between S_1_ and T_1_ states, both *p*‐MOTPPIO and *p*‐MOTBPIO show large energy gaps over 1 eV and small SOC values below 0.1 cm^−1^, indicating that their ISC processes from S_1_ to T_1_ states are inefficient. By contrast, the ISC processes from S_1_ to T_2_ states are more feasible, as evidenced by the smaller energy gaps and larger SOC values. Especially, *p*‐MOTBPIO has the larger SOC value (1.007 cm^−1^) and smaller energy gap (0.5 eV) between S_1_ and T_2_ states than *p*‐MOTPPIO, accounting for its higher ROS generation capability.

### 2.6 In Situ Photocyclization and Lipid Droplet “Light Up”

**Figure 6:**
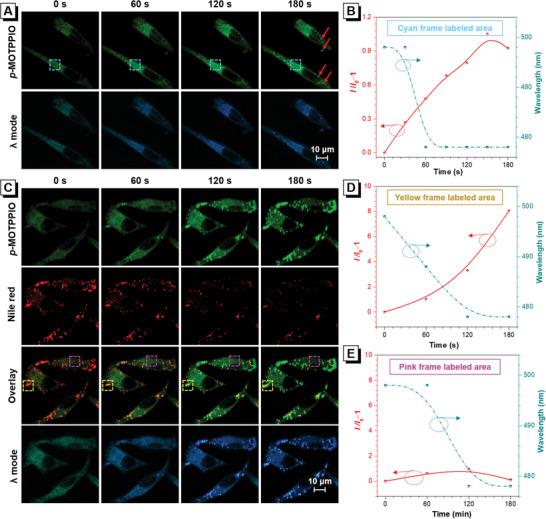
A) CLSM images in channel and λ modes of HeLa cells incubated with *p*‐MOTPPIO (2 µm) for 1 h at continuous irradiation by 405 nm laser with 5% power. B) Plots of relative signal intensity and PL peak versus irradiation time in cyan frame labeled area. C) CLSM images in channel and λ modes of HeLa cells with oleic acid pretreatment incubated with *p*‐MOTPPIO (5 µm) and Nile red (5 µg mL^−1^) (the latter with brightness correction using ZEN) for 1 h at continuous irradiation by 405 nm laser with 5% power. Plots of relative signal intensity and PL peak versus irradiation time in D) yellow frame labeled area and E) pink frame labeled area.

The potential of *p*‐MOTPPIO and *p*‐MOTBPIO in biological application is explored. The excessive hydrophobicity makes *p*‐MOTPPIO and *p*‐MOTBPIO form nanoparticles in aqueous environments (Figure S21, Supporting Information). The intracellular uptake process is first studied by confocal laser scanning microscopy (CLSM), in which the HeLa cell line is adopted as the model. *p*‐MOTPPIO and *p*‐MOTBPIO can quickly penetrate cytomembrane upon incubating for 30 min at 37 °C, showing vivid fluorescence peaking at 498 and 478 nm, respectively (Figures S22 and S23, Supporting Information). The transmembrane process can occur even under 4 °C. This manifests that *p*‐MOTPPIO and *p*‐MOTBPIO enter cells mainly via free diffusion pathway without the participation of energy and carrier protein, due to the adequate hydrophobicity. As follows, in situ intracellular photoactivation behavior of *p*‐MOTPPIO in different concentrations is studied under continuous excitation and sequential scanning by using a 405 nm laser with 5% power. As depicted in **Figure** **6**A,B; Figures S24 and S25 (Supporting Information), the fluorescence in HeLa cells becomes slightly increased and blue‐shifted from 498 to 478 nm under irradiation, which is attributed to the photoconversion of *p*‐MOTPPIO to *p*‐MOTBPIO. At ≈150 s, the fluorescence intensity reaches the peak, indicating the photocyclization is complete (Figure 6B).

It is noteworthy that scattered bright spots appear with the extension of irradiation time (red arrow in Figure 6A). Considering these newly emerged spots may locate at LD region,^[^ ^29^ ^]^ to make the bright spots more pronounced, oleic acid is applied to promote LD generation in HeLa cells. With 100 µM oleic acid pretreatment for 6‒12 h, only green fluorescence from *p*‐MOTPPIO is observed with uniform intensity throughout the cytoplasm when the light is firstly applied, and plenty of blue spots from the photocyclization product, *p*‐MOTBPIO, are clearly visualized and get brighter by increasing irradiation time (Figure S26, Supporting Information). As follows, colocalization imaging of *p*‐MOTPPIO with Nile Red (a commercial LD probe) are conducted to verify the cellular location of “light up” site (Figure 6C). As expected, before photoactivation, fluorescence signals from *p*‐MOTPPIO barely overlap with those of Nile Red, while after photoactivation, the bright spots are well overlapped with those of Nile Red, disclosing the “light up” areas are associated with LDs. Here, the signals from Nile Red are gradually decreased by increasing irradiation time due to its poor photostability. It is noted that the signal intensity in LD area (e.g., yellow frame in Figure 6C) shows nearly 9‐fold enhancements after irradiation for 3 min (Figure 6D), while that in other cytoplasm area (e.g., pink frame in Figure 6C) only shows less than 2‐fold enhancements and slightly decreases along with the increase of irradiation time (Figure 6E). The phenomenon of enhanced florescence in LD area and weakened florescence in other cytoplasm area is possibly caused by location transfer of *p*‐MOTBPIO from other cytoplasm area to LDs. Using *p*‐MOTBPIO to stain HeLa cells pretreated with oleic acid, uniform blue fluorescence signals are found throughout the cytoplasm area before irradiation, while lots of scattered spots appear and become brighter due to redistribution and further accumulation of *p*‐MOTBPIO under irradiation, confirming that the “light up” phenomenon of LD is a light‐driven process. In addition, similar phenomena are also observed in LD‐abundant A549 cells (Figure S27, Supporting Information), indicating *p*‐MOTPPIO and *p*‐MOTBPIO could universally “light up” cells under irradiation.

### 2.7 ROS Generation and PDT In Vitro

**Figure 7:**
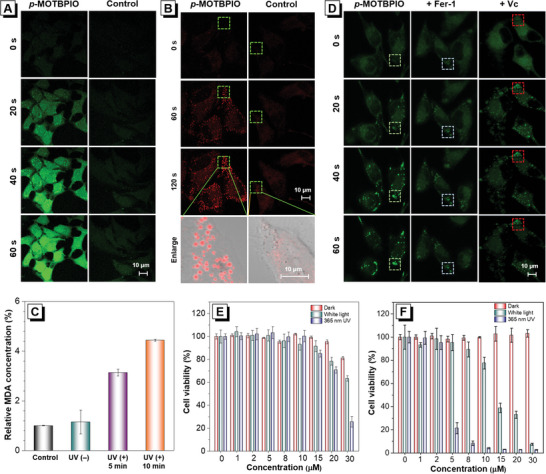
CLSM images of A) general ROS and B) lipid peroxides generation in HeLa cells incubated with *p*‐MOTBPIO (5 µm) for 1 h using DCFH‐DA and Liperfluo as indicator, respectively, under continuous irradiation by 405 nm laser with 5% power. C) Relative MDA concentration in HeLa cells incubated with *p*‐MOTBPIO (5 µm) for 1 h and then treated by different conditions. D) CLSM images of HeLa cells with oleic acid pretreatment with *p*‐MOTBPIO (5 µm) for 1 h with or without Fer‐1 or Vc at continuous irradiation by 405 nm laser with 5% power. Cell viability of HeLa cells after treatment with a range of concentrations of E) *p*‐MOTPPIO and F) *p*‐MOTBPIO under different conditions (white light: 20 mW cm^−2^, 10 min; 365 nm UV: 10 mW cm^−2^, 1 min).

Considering the ROS generation ability of *p*‐MOTBPIO, it is envisioned that the photoactivated LD “light up” phenomenon may cause cell damage because of ROS. To verify this hypothesis, in situ ROS generation capability of *p*‐MOTBPIO in cells is examined by using 2,7‐dichlorodihydro fluorescein diacetate (DCFH‐DA) as a universal indicator. Intensified fluorescence signals are observed in *p*‐MOTBPIO‐stained HeLa cells under continuous irradiation, while the signals in control group maintain constant (**Figure** **7**A and Figure S28A, Supporting Information), signifying the efficient intracellular ROS generation ability of *p*‐MOTBPIO.

To the best of our knowledge, intracellular ROS generation is able to trigger lipid peroxidation, producing lipid peroxyl radicals, hydroperoxides, and various oxidation by‐products.^[^ ^30^ ^]^ Based on this, Liperfluo is utilized to in situ visualize the lipid peroxide generation in cells, and Lipid Peroxidation MDA (malondialdehyde) Assay Kit is employed to detect the secreted lipid peroxides.^[^ ^31^ ^]^ As shown in Figure 7B and Figure S28B (Supporting Information), HeLa cells pretreated by oleic acid are flooded with signals from Liperfluo after continuous irradiation. As shown in Figure 7C, under UV irradiation, the MDA concentration of *p*‐MOTBPIO‐treated group is enhanced by nearly 5‐fold higher than that of control group. Both results confirm the formation of lipid peroxides, which should be attributed to the PDT effect of *p*‐MOTBPIO. Furthermore, a radical scavenger of vitamin C (Vc) and an antioxidant agent of Ferrostatin‐1 (Fer‐1) are added into *p*‐MOTBPIO‐treated cells^[^ ^32^ ^]^ to remove the generated ROS and inhibit lipid peroxidation. The LD “light up” process is found to be suppressed to some extent by Vc and Fer‐1, in comparison with that in cells treated with *p*‐MOTBPIO only (Figure 7D; Figures S29, S30, and S31, Supporting Information), further validating that the formation of lipid peroxides results in the accumulation of *p*‐MOTBPIO in LDs and thus fluorescence “light up”. Meanwhile, triglyceride is utilized to simulate the environment of lipid droplets, and it is found that the fluorescence intensity is directly proportional to the concentration of *p*‐MOTBPIO within a certain concentration range (Figure S32, Supporting Information).

Next, to assess the biosafety and anticancer potential of *p*‐MOTPPIO and *p*‐MOTBPIO, their cytotoxicity is evaluated by 3‐(4,5‐dimethyl‐2‐thiazolyl)−2,5‐diphenyltetrazolium bromide (MTT) assay under both dark and light conditions. As shown in Figure 7E and Figure S33 (Supporting Information), *p*‐MOTPPIO and *p*‐MOTBPIO show negligible cytotoxicity toward HeLa, while causing slight damage L929 cells (normal cells) under high concentration after 24 h incubation in dark condition. When white light irradiation (20 mW cm^−2^, 10 min) is applied on cells after the treatment of *p*‐MOTPPIO or *p*‐MOTBPIO for 1 h, the cell viability is recorded over 60% for *p*‐MOTPPIO even with a high concentration of 30 µM, while for *p*‐MOTBPIO, ≈40% cell viability remains at 15 µM and <10% cell viability remains at 30 µM, revealing that *p*‐MOTBPIO holds obvious cytotoxicity toward HeLa cells, owing to its excellent ROS generation ability (Figure 7F). It is demonstrated that *p*‐MOTBPIO is a promising candidate as an efficient PS for anticancer PDT. Considering the limited absorption of *p*‐MOTPPIO and *p*‐MOTBPIO in white light region, UV light irradiation (365 nm, 10 mW cm^−2^, 1 min) is then applied to uncover their biological application potential. Upon UV light irradiation, *p*‐MOTPPIO acquires a decent PDT effect via the formation of *p*‐MOTBPIO, which shows an improved killing effect toward HeLa cells. Although UV light irradiation has some degree of damage on cells, these results just suggest that *p*‐MOTPPIO is promising as a core structure to develop photoactivated and self‐reported PSs for anticancer PDT by further molecular engineering.

## 3 Conclusion

In summary, a series of new AIE‐active TPPIO derivatives with diverse substituents are designed and prepared. Experimental and theoretical studies prove that these TPPIO derivatives can undergo free radical‐mediated [6π] photocyclization to generate novel TBPIO derivatives in solutions with the participation of trace oxygen. It is noteworthy that the substituents can greatly influence the photocyclization efficiency, and the electron‐withdrawing substituents are beneficial to the photocyclization of *para‐*substituted TPPIO derivatives, while electron‐donating substituents are good for the photocyclization of *meta‐*substituted ones. Additionally, TPPIO and TBPIO derivatives show distinct photophysical/photochemical properties. Taking *p*‐MOTPPIO and *p*‐MOTBPIO for comparation, the former owns AIE property but barely generates ROS, while the latter experiences ACQ but exhibits efficient Type I ROS generation capacity. Interestingly, *p*‐MOTPPIO and *p*‐MOTBPIO show high cellular permeability and low dark cytotoxicity. The in vitro results demonstrate that *p*‐MOTPPIO can efficiently undergo in situ photocyclization in living cells to activate PDT upon continuous laser excitation. Meanwhile, its efficient intracellular ROS generation can trigger lipid peroxidation, which results in the accumulation of *p*‐MOTBPIO in LDs and further leads to fluorescence “light up”. Moreover, the ROS‐based lipid peroxidation induced by *p*‐MOTBPIO‐mediated PDT has a high potential as a precursor of the ferroptosis inducer for antitumor therapy. Based on these interesting results, it is believed that this new photocyclization reaction can greatly broaden synthetic routes and application scenarios of the optoelectronically and biologically useful P≐O‐containing π‐conjugated systems.

## Conflict of Interest

The authors declare no conflict of interest.

## Supporting information

Supporting Information

Supporting Information

## Data Availability

The data that support the findings of this study are available from the corresponding author upon reasonable request.
