# Supplementary material for: Free Radical‐Mediated Photocyclization of Triphenylphosphindole Oxides for Photoactivated and Self‐Reported Lipid Peroxidation
Source: Adv Sci (Weinh). 2023 Oct 23;10(35):2305516. doi: 10.1002/advs.202305516 (PMC10724397; doi:10.1002/advs.202305516)
Supplement: Supplementary file 1 — Supporting Information [file ADVS-10-2305516-s002.pdf]

ADVANCED SCIENCE
Open Access

# Supporting Information

for *Adv. Sci.*, DOI 10.1002/advs.202305516

## Free Radical-Mediated Photocyclization of Triphenylphosphindole Oxides for Photoactivated and Self-Reported Lipid Peroxidation

*Jianqing Li, Zeyan Zhuang, Jingjing Guo, Xiaobin Dong, Junyi Gong, Ben Zhong Tang and Zujin Zhao**

# Supporting Information

## 1 Materials and Instruments

### 1.1 Materials

All the solvents and reagents used in this work were of analytical grade. Phosphate buffered saline (PBS), fetal bovine serum (FBS), penicillin and streptomycin were purchased from Thermo Fisher Scientific. Dulbecco's Modified Essential Medium (DMEM) was purchased from Gibco (Life Technologies). 3-(4,5-Dimethyl-2-thiazolyl)-2,5-diphenyl-2H-tetrazolium bromide (MTT) was purchased from J&K Scientific Ltd. 2,7-Dichlorodihydrofluorescein diacetate (DCFH-DA) and 9,10-anthracenediyl-bis(methylene) dimalonic acid (ABDA) were purchased from E. MerckChemical Co. Ltd. Lipid Peroxidation Malonaldehyde (MDA) Assay Kit were purchased from Beyotime Biotechnology. 5,5-Dimethyl-1-pyrroline N-oxide (DMPO), Ferrostatin-1 (Fer-1), Nile Red and Dihydrorhodamine 123 (DHR123) were purchased from MedChemExpress Co., Ltd. Commercial dyes ER-Tracker Red was purchased from Thermo Thermo Fisher Scientific Co., Ltd. Liperfluo-Lipid Peroxidation Probe was purchased from Dojindo Co., Ltd. Rose Bengal (RB), Vitamin C (Vc), and oleic acid (OA) were purchased from Shanghai Aladdin Biochemical Technology Co., LTD. HeLa and A549 cells were obtained from the cell culture center of the Institute of Basic Medical Sciences, Chinese Academy of Medical Science. All other chemicals and reagents were purchased from commercial companies and used as received without further purification.

### 1.2 Instruments

$^{1}H$ and $^{13}C$ NMR spectra were tested on a Bruker AV 400 or 500 spectrometers in deuterated methylene dichloride, chloroform, or dimethyl sulfoxide using tetramethylsilane (TMS; $\delta = 0$) as an internal reference at room temperature. Electron paramagnetic resonance (EPR) measurements were carried out on Bruker ELEXSYS-II E500 in X-band. The 365 nm UV light irradiation energy density was determined by 365 nm irradiatometer. High-resolution

mass spectra (HRMS) were recorded on Agilent1290/ Bruker maxis impact mass spectrometer operating in positive mode and the Waters G2-XS QT of MS mass spectrometer. The purity analysis was tested on Waters 2489 high performance liquid chromatograph (HPLC). The LC-Forte/R Multiple preparative HPLC was used to separate and purify parts of photoproducts. Single-crystal X-ray diffraction (XRD) data were collected from a Rigaku XtaLAB P200 FR-X: four-circle Kappa single diffractometer. UV-vis absorption spectra were measured on a SHIMADZU UV-2600 spectrophotometer. Photoluminescence (PL) spectra were recorded on a Horiba Fluoromax-4 fluorescence spectrophotometer. Fluorescence quantum yields were measured using a Hamamatsu absolute PL quantum yield spectrometer C11347 Quantaurus_QY. Particle size analysis was performed on a Malvern Zetasizer Nano-S90. Confocal laser scanning microscopy (CLSM) images were obtained on a Zeiss LSM7 DUO Laser Scanning Confocal Microscope. Automated cell counter (Countess II) was employed for cell counting. The cell viability analysis was collected by using a microplate reader (Tecan Infinite M200PRO) at a wavelength of 570 nm. The white light source was a CXE-350 xenon lamp fiber illumination system produced by Photoelectronic Instrument Factory of Beijing Normal University. The irradiation light energy density was measured using a FZ-A irradiatometer produced by Photoelectronic Instrument Factory of Beijing Normal University. Unless otherwise stated, the power density of 365 nm UV is about 40 mW $cm^{-2}$.

## 2 Methods and Experimental Procedures

### 2.1 Syntheses and characterization

**1,2,3-Triphenylphosphindole 1-oxide (TPPIO), 2,3-di([1,1'-biphenyl]-4-yl)-1-phenylphosphindole 1-oxide (*p*-PhTPPIO)** and **2,3-di([1,1'-biphenyl]-3-yl)-1-phenylphosphindole 1-oxide (*m*-PhTPPIO)** were synthesized according the literature methods.[1]

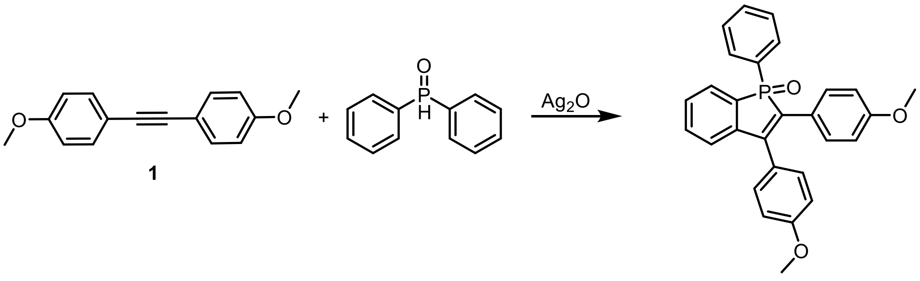


**2,3-Bis(4-methoxyphenyl)-1-phenylphosphindole 1-oxide (*p*-MOTPPIO):** Compound **1** was synthesized according to the literature method.[2] A mixture of compound **1** (2.38 g, 10 mmol), diphenylphosphine oxide (4.04 g, 20 mmol) and $Ag_2O$ (4.62 g, 20 mmol) in DMF (125 mL) was stirred at 100 $^{o}$C for 10 h under nitrogen. After cooling to room temperature and solvent evaporation, the mixture was extracted successively with dichloromethane and dried over anhydrous sodium sulfate. Then, the solvent was evaporated under reduced pressure. The residue was purified by silica-gel column chromatography and then recrystallization. *p*-MOTPPIO was obtained as yellowish-green solid in 87% yield. $^{1}$H NMR (500 MHz, $CD_2Cl_2$) $\delta$ (TMS, ppm): 7.76–7.71 (m, 2H), 7.64–7.61 (m, 1H), 7.49–7.47 (m, 2H), 7.42–7.38 (m, 3H), 7.29 (d, $J$ = 8.3 Hz, 2H), 7.24 (dd, $J$ = 9.2, 3.0 Hz, 1H), 7.20 (dd, $J$ = 8.5, 1.2 Hz, 2H), 6.99 (d, $J$ = 8.9 Hz, 2H), 6.66 (d, $J$=8.8 Hz, 2H), 3.85 (s, 3H), 3.69 (s, 3H). $^{13}$C NMR (100 MHz, $CD_2Cl_2$) $\delta$ (TMS, ppm): 159.91, 159.20, 148.22 (d, $J$ = 21.5 Hz), 144.13 (d, $J$ = 26.9 Hz), 133.19 (d, $J$ = 96.4 Hz), 132.78 (d, $J$ = 2.1 Hz), 132.00 (d, $J$ = 2.9 Hz) 131.72, 130.85 (d, $J$ = 10.4 Hz), 130.71 (d, $J$ = 98.1 Hz), 130.48, 130.32 (d, $J$ = 5.9 Hz), 128.79 (d, $J$ = 12.1 Hz), 128.72 (d, $J$ = 10.5 Hz) 128.50 (d, $J$ = 9.6 Hz), 126.45 (d, $J$ = 15.0 Hz), 125.44 (d, $J$ = 10.3 Hz), 123.76 (d, $J$ = 10.9 Hz), 114.40, 113.69, 55.28, 55.07. HRMS: $m/z$ [M + $H^+$] found, 439.1451 ($C_{28}H_{24}O_3P$, calcd., 439.1463).

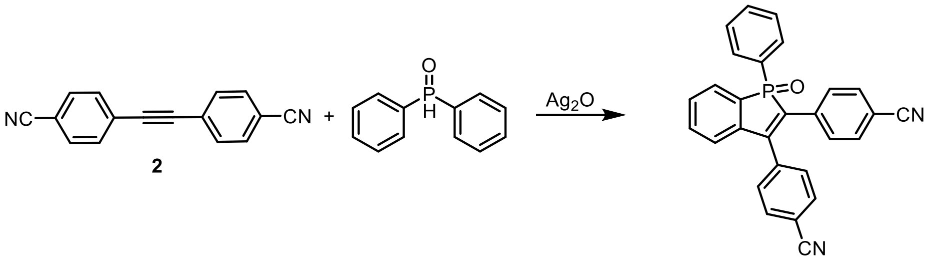


**4,4'-(1-Oxido-1-phenylphosphindole-2,3-diyl)dibenzonitrile (*p*-CNTPPIO)**: Compound **2** was synthesized according to the literature method.[3] The procedure was analogous to that described for *p*-MOTPPIO. White solid, yield 73%. $^1$H NMR (400 MHz, $CDCl_3$) $\delta$ (TMS, ppm): 7.79–7.75 (m, 3H), 7.73–7.67 (m, 2H), 7.56–7.52 (m, 2H), 7.51–7.48 (m, 1H), 7.46–7.41 (m, 6H), 7.29 (d, *J* = 7.6 Hz, 2H), 7.16 (dd, *J* = 7.5, 2.9 Hz, 1H). $^{13}$C NMR (100 MHz, $CDCl_3$) $\delta$ (TMS, ppm): 149.89 (d, *J* = 21.4 Hz ), 142.10 (d, *J* = 25.6 Hz), 138.29 (d, *J* = 14.4 Hz), 136.91 (d, *J* = 9.6 Hz), 134.88 (d, *J* = 93.0 Hz), 133.49 (d, *J* = 2.0 Hz), 133.03, 132.92 (d, *J* = 2.9 Hz), 132.31, 131.62 (d, *J* = 105.9 Hz), 130.85 (d, *J* = 10.7 Hz), 130.37 (d, J = 10.7 Hz), 129.84, 129.77, 129.41 (d, *J* = 5.4 Hz), 129.22 (d, *J* = 12.5 Hz), 128.35 (d, *J* = 100.7 Hz), 124.17 (d, *J* = 10.6 Hz), 118.26, 118.01, 113.29, 111.96. HRMS: *m/z* [M + H$^+$] found, 429.1148 ($C_{28}H_{18}N_2OP$, calcd., 429.1157).

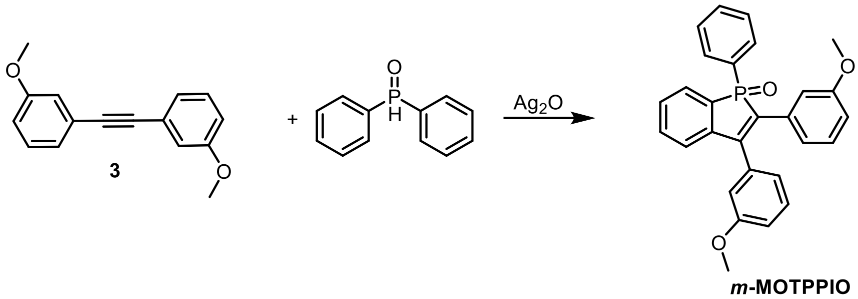


**2,3-Bis(3-methoxyphenyl)-1-phenylphosphindole 1-oxide (*m*-MOTPPIO):** Compound **3** was synthesized according to the literature method.[4] The procedure was analogous to that described for *p*-MOTPPIO. White solid, yield 73%. $^1$H NMR (500 MHz, $CD_2Cl_2$) $\delta$ (TMS, ppm): 7.78–7.74 (m, 2H), 7.67–7.64 (m, 1H), 7.51–7.48 (m, 2H), 7.44–7.38 (m, 4H), 7.24 (dd, *J* = 7.7, 2.9 Hz, 1H), 7.05 (m, 1H), 6.99–6.94 (m, 2H), 6.92–6.88 (m, 2H), 6.72 (d, *J* = 1.4 Hz,

1H), 6.69–6.67 (m, 1H), 3.76 (s, 3H), 3.50 (s, 3H). $^{13}$C NMR (100 MHz, $CD_2Cl_2$) $\delta$ (TMS, ppm): 160.59, 159.66, 150.38 (d, $J$ = 21.2 Hz), 144.06 (d, $J$ = 26.6 Hz), 136.17 (d, $J$ = 14.7 Hz), 134.53 (d, $J$ = 94.8 Hz), 134.52 ($J$ = 10.1 Hz), 133.35 (d, $J$ = 2.0 Hz), 133.18, 132.59 (d, $J$ = 2.8 Hz), 132.29, 132.13, 131.32 (d, $J$ = 10.5 Hz), 130.57, 130.26, 129.60 (d, $J$ =10.6 Hz), 129.63, 129.28 (d, $J$ = 12.1 Hz), 129.06 (d, $J$ = 9.6 Hz), 124.59 (d, $J$ = 10.6 Hz), 121.77 (d, $J$ = 5.3 Hz), 121.58, 114.86, 114.58, 114.18 (d, $J$ =5.6 Hz), 55.74, 55.25. HRMS: *m/z* [M + $H^+$] found, 439.1456 ($C_{28}H_{24}O_3P$, calcd., 439.1463).

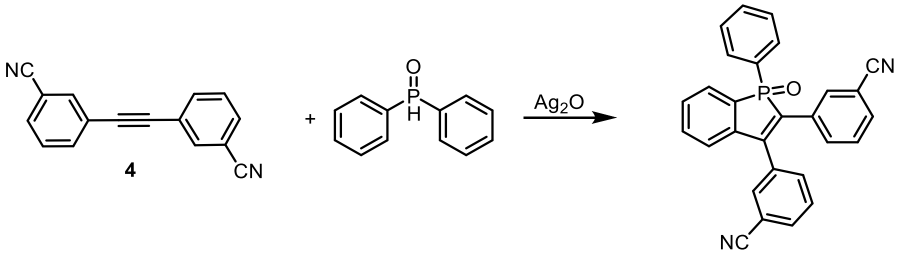


**3,3'-(1-Oxido-1-phenylphosphindole-2,3-diyl) dibenzonitrile (*m*-CNTPPIO):** Compound **4** was synthesized according to the literature method.[5] The procedure was analogous to that described for *p*-MOTPPIO. White solid, yield 73%. $^1$H NMR (400 MHz, $CD_2Cl_2$) $\delta$ (TMS, ppm): 7.78–7.66 (m, 5H), 7.62–7.49 (m, 5H), 7.48–7.41 (m, 5H), 7.28 (m, 1H), 7.20 (dd, $J$ = 7.6, 3.1 Hz, 1H). $^{13}$C NMR (100 MHz, $CD_2Cl_2$) $\delta$ (TMS, ppm): 149.62 (d, $J$ = 21.3 Hz), 142.66 (d, $J$ = 25.6 Hz), 135.07 (d, $J$ = 14.5 Hz), 134.99 (d, $J$ = 93.6 Hz), 134.10 (d, $J$ = 9.8 Hz), 133.96, 133.78 (d, $J$ = 2.2 Hz), 133.53 (d, $J$ = 5.0 Hz), 133.22, 133.13 (d, $J$ = 2.8 Hz), 132.88, 132.25 (d, $J$ = 105.6 Hz), 132.48 (d, $J$ = 5.3 Hz), 132.15, 131.27 (d, $J$ = 10.6 Hz), 130.68, 130.58 (d, $J$ = 10.7 Hz), 129.90, 129.81 (d, $J$ = 9.7 Hz), 129.59, 129.47, 129.22 (d, $J$ = 100.2 Hz), 124.58 (d, $J$ = 10.6 Hz), 118.44 (d, $J$ = 5.9 Hz), 114.03, 113.32. HRMS: *m/z* [M + $H^+$] found, 429.1152 ($C_{28}H_{18}N_2OP$, calcd., 429.1157).

**3,6-Dimethoxy-9-phenyltribenzo[b,e,g]phosphindole 9-oxide (*p*-MOTBPIO):** *p*-MOTPPIO (220 mg, 0.5 mmol) in methanol (100 mL) in the reaction bulb was bubbled with $N_2$ for 30 min. Afterward, the mixture was irradiated with 365 nm UV lamp under $N_2$ flow.

After irradiation, the resulting solution was evaporated under reduced pressure. The photoproduct was purified by silica-gel column chromatography and then recrystallization. *p*-MOTBPIO was obtained as yellow solid in 72.1% yield. $^1$H NMR (400 MHz, $CD_2Cl_2$) $\delta$ (TMS, ppm): 8.93 (d, $J$ = 9.3 Hz, 1H), 8.48 (dd, $J$ = 7.9, 3.4 Hz, 1H), 8.16 (d, $J$ = 8.1 Hz, 1H), 8.10 (d, $J$ = 2.7 Hz, 1H), 7.98 (s, 1H), 7.78–7.73 (m, 1H), 7.71–7.66 (m, 3H), 7.48–7.41 (m, 3H), 7.38–7.33 (m, 2H), 7.21 (dd, $J$ = 8.9, 2.5 Hz, 1H), 4.07 (s, 3H), 3.98 (s, 3H). $^{13}$C NMR (100 MHz, $CD_2Cl_2$) $\delta$ (TMS, ppm): 159.86, 159.55, 143.15 (d, $J$ = 23.1 Hz), 137.38 (d, $J$ = 20.1 Hz), 135.62 (d, $J$ = 2.4 Hz), 134.58, 133.43 (d, $J$ = 2.0 Hz), 132.47, 132.42 (d, $J$ = 2.9 Hz), 131.78 (d, $J$ = 8.7 Hz), 132.18 (d, $J$ = 8.7 Hz), 131.37 (d, $J$ = 10.8 Hz), 129.98 (d, $J$ = 9.8 Hz), 129.18 (d, $J$ = 12.3 Hz), 128.84 (d, $J$ = 11.1 Hz), 128.58 (d, $J$ =5.5 Hz), 127.88, 127.64 (d, $J$ = 103.4 Hz), 125.63 (d, $J$ =10.7 Hz), 124.67 (d, $J$ = 9.2 Hz), 123.27 (d, $J$ =12.3 Hz), 118.13, 117.44, 106.21, 105.60, 55.99 (d, $J$ = 3.3 Hz). HRMS: *m/z* [M + $H^+$] found, 437.1291 ($C_{28}H_{22}O_3P$, calcd., 437.1306).

**3,6,9-Triphenyltribenzo[b,e,g]phosphindole 9-oxide (*p*-PhTBPIO):** The procedure was analogous to that described for *p*-MOTBPIO. White solid, yield 80.3%. $^1$H NMR (500 MHz, $CD_2Cl_2$) $\delta$ (TMS, ppm): 9.11–9.08 (m, 2H), 8.97 (s, 1H), 8.60 (d, $J$ = 4.7 Hz, 1H), 8.34 (d, $J$ = 8.4 Hz, 1H), 8.08 (dd, $J$ = 8.6, 1.9 Hz, 1H), 7.88 (d, $J$ = 7.5 Hz, 2H), 7.83–7.80 (m, 2H), 7.77–7.72 (m, 5H), 7.57 (m, 2H), 7.52–7.46 (m, 5H), 7.43–7.36 (m, 3H). $^{13}$C NMR (100 MHz, $CD_2Cl_2$) $\delta$ (TMS, ppm): 141.62, 141.03 (d, $J$ = 44.8 Hz), 141.07, 133.66 (d, $J$ = 1.6 Hz), 132.63 (d, $J$ = 3.0 Hz), 131.45 (d, $J$ = 11.0 Hz), 130.22 (d, $J$ = 11.0 Hz), 129.52, 129.40, 129.24, 128.42 (d, $J$ = 20.9 Hz), 128.04, 127.98, 127.90, 127.60, 127.29, 126.94, 126.16 (d, $J$ = 10.9 Hz), 122.63, 121.98. HRMS: *m/z* [M + $H^+$] found, 529.1716 ($C_{38}H_{26}OP$, calcd., 529.1721).

**9-Phenyltribenzo[b,e,g]phosphindole-3,6-dicarbonitrile 9-oxide (*p*-CNTBPIO):** The procedure was analogous to that described for *p*-MOTBPIO. Due to the poor solubility, *p*-CNTBPIO was precipitated in methanol and collected directly by filtration. White solid, yield

90.5%. $^{1}$H NMR (400 MHz, $CD_2Cl_2$) $\delta$ (TMS, ppm): 9.12–9.09 (m, 2H), 8.97 (s, 1H), 8.51 (dd, $J$ = 8.1, 3.6 Hz, 1H), 8.42 (d, $J$ = 8.4 Hz, 1H), 8.07 (dd, $J$ = 8.7, 1.68 Hz, 1H), 7.88–7.77 (m, 3H), 7.69–7.66 (m, 2H), 7.61–7.58 (m, 1H), 7.51–7.49 (m, 1H), 7.41–7.37 (m, 2H). $^{13}$C NMR (100 MHz, $CD_2Cl_2$) $\delta$ (TMS, ppm): 141.82 (d, $J$ = 19.7 Hz), 141.23 (d, $J$ = 21.9 Hz), 134.92 (d, $J$ = 106.2 Hz), 134.10 (d, $J$ = 2.0 Hz), 133.90, 133.15 (d, $J$ = 2.9 Hz), 132.97 (d, $J$ = 0.6 Hz), 132.90, 132.16 (d, $J$ = 8.7 Hz), 131.38 (d, $J$ = 10.8 Hz), 130.83, 130.71 (d, $J$ = 2.5 Hz), 130.67, 130.61, 130.41, 129.84 (d, $J$ = 7.9 Hz), 129.57, 129.52, 129.45, 128.89, 128.26 (d, $J$ = 5.4 Hz), 127.67, 126.54 (d, $J$ = 10.6 Hz), 118.75 (d, $J$ = 26.6 Hz), 113.38, 112.41. HRMS: $m/z$ [M + H$^{+}$] found, 427.0987 ($C_{28}H_{18}N_2OP$, calcd., 427.1000).

**9-Phenyltribenzo[b,e,g]phosphindole 9-oxide (TBPIO):** The procedure was analogous to that described for *p*-MOTBPIO. White solid, yield 92%. $^{1}$H NMR (400 MHz, $CD_2Cl_2$) $\delta$ (TMS, ppm): 9.09–8.94 (m, 1H), 8.90–8.78 (m, 1H), 8.70 (d, $J$ = 8.4 Hz, 1H), 8.57 (dd, $J$ = 8.0, 3.5 Hz, 1H), 8.27 (d, $J$ = 8.0 Hz, 1H), 7.88–7.75 (m, 3H), 7.75–7.62 (m, 4H), 7.60–7.53 (m, 1H), 7.46 (m, 2H), 7.35 (m, 2H). $^{13}$C NMR (100 MHz, $CD_2Cl_2$) $\delta$ (TMS, ppm): 142.41 (d, $J$ = 23.1 Hz), 139.44 (d, $J$ = 19.6 Hz), 134.80 (d, $J$ = 105.4 Hz), 133.82 (d, $J$ = 2.0 Hz), 133.14 (d, $J$ = 2.1 Hz), 132.15 (d, $J$ = 2.9 Hz), 131.65, 130.98 (d, $J$ = 10.7 Hz), 130.75 (d, $J$ = 8.3 Hz), 130.66 (d, $J$ = 3.2 Hz), 129.75 (d, $J$ = 9.8 Hz), 129.21 (d, $J$ = 8.8 Hz), 129.03, 128.92, 128.89, 128.76, 128.63, 128.07, 127.89 (d, $J$ = 3.9 Hz), 127.53, 126.69 (d, $J$ = 5.5 Hz), 125.88, 125.83, 125.72, 124.04, 123.19. HRMS: $m/z$ [M + Na$^{+}$] found, 399.0928 ($C_{26}H_{17}OPNa$, calcd., 399.0915).

**4,5-Dimethoxy-9-phenyltribenzo[b,e,g]phosphindole 9-oxide (*m*-MOTBPIO-1):** The procedure was analogous to that described for *p*-MOTBPIO. The photoproduct was firstly purified by silica-gel column chromatography. HPLC with pure acetonitrile (MeCN) as mobile phase was then used for further purification. Yellow solid, yield 1.4%. $^{1}$H NMR (400 MHz, $CD_2Cl_2$) $\delta$ (TMS, ppm): 8.47 (dd, $J$ = 8.3, 4.5 Hz, 2H), 7.80–7.63 (m, 6H), 7.50–7.42 (m, 3H), 7.38–7.27 (m, 3H), 7.11 (d, $J$ = 8.0 Hz, 1H), 4.04 (s, 3H), 4.02 (s, 3H). $^{13}$C NMR

(100 MHz, $CD_2Cl_2$) $\delta$ (TMS, ppm): 157.95, 157.91 (d, $J$ = 2.0 Hz), 142.21 (d, $J$ = 23.0 Hz), 139.79 (d, $J$ = 20.0 Hz), 134.11, 132.91 (d, $J$ = 1.0 Hz), 132.09 (d, $J$ = 3.0 Hz), 131.27 (d, $J$ = 9.0 Hz), 131.08 (d, $J$ = 11.0 Hz), 130.52, 130.03, 129.93, 129.79, 128.78 (d, $J$ = 13.0 Hz), 128.71 (d, $J$ = 11.0 Hz), 128.14, 127.62, 125.48 (d, $J$ = 10.0 Hz), 123.16, 118.86 (d, $J$ = 9.0 Hz), 117.59 (d, $J$ = 5.0 Hz), 116.51, 110.19, 109.37, 55.92, 55.80. HRMS: *m/z* [M + $H^+$] found, 437.1320 ($C_{28}H_{22}O_3P$, calcd., 437.1307).

**4,7-Dimethoxy-9-phenyltribenzo[b,e,g]phosphindole 9-oxide (*m*-MOTBPIO-2):** The procedure was analogous to that described for *p*-MOTBPIO. The photoproduct was firstly purified by silica-gel column chromatography. HPLC with pure MeCN as mobile phase was then used for further purification. Yellow solid, yield 25.4%. $^1$H NMR (400 MHz, $CD_2Cl_2$) $\delta$ (TMS, ppm): 9.81 (d, $J$ = 9.5 Hz, 1H), 8.54 (dd, $J$ = 8.1, 3.4 Hz, 1H), 8.39 (d, $J$ = 2.8 Hz, 1H), 7.92 (d, $J$ = 8.0 Hz, 1H), 7.80 (dd, $J$ = 10.7, 7.2 Hz, 1H), 7.74–7.65 (m, 3H), 7.51–7.41 (m, 4H), 7.36 (m, 2H), 7.17 (d, $J$ = 7.0 Hz, 1H), 4.11 (s, 3H), 4.07 (s, 3H). $^{13}$C NMR (100 MHz, $CD_2Cl_2$) $\delta$ (TMS, ppm): 158.08, 157.91 (d, $J$ = 2.0 Hz), 142.16 (d, $J$ = 23.0 Hz), 139.50 (d, $J$ = 20.0 Hz), 134.32 (d, $J$ = 6.0 Hz), 133.09 (d, $J$ = 2.0 Hz), 132.10 (d, $J$ = 3.0 Hz), 131.74, 130.99, 130.93, 130.88, 130.73, 130.44 (d, $J$ = 9.0 Hz), , 129.97, 129.83, 129.73, 129.70, 128.96, 128.85, 128.73, 128.23 (d, $J$ = 2.0 Hz), 127.03, 125.36 (d, $J$ = 11.0 Hz), 121.24 (d, $J$ = 9.0 Hz), 119.39 (d, $J$ = 6.0 Hz), 117.31, 109.68, 107.00, 55.78, 55.49. HRMS: *m/z* [M + $H^+$] found, 437.1313 ($C_{28}H_{22}O_3P$, calcd., 437.1307).

**2,5-Dimethoxy-9-phenyltribenzo[b,e,g]phosphindole 9-oxide (*m*-MOTBPIO-3):** The procedure was analogous to that described for *p*-MOTBPIO. The photoproduct was firstly purified by silica-gel column chromatography. HPLC with pure MeCN as mobile phase was then used for further purification. Yellow solid, yield 8.7%. $^1$H NMR (400 MHz, $CD_2Cl_2$) δ (TMS, ppm): δ 9.60 (d, $J$ = 9.5 Hz, 1H), 8.62 (d, $J$ = 8.3 Hz, 1H), 8.53 (dd, $J$ = 7.8, 3.0 Hz, 1H), 7.87–7.58 (m, 6H), 7.42 (m, 5H), 7.22 (dd, $J$ = 9.6, 2.8 Hz, 1H), 4.15 (s, 3H), 3.83 (s, 3H). $^{13}$C NMR (100 MHz, $CD_2Cl_2$) δ (TMS, ppm): 158.63, 157.99, 142.72 (d, $J$ = 22.0 Hz),

139.85 (d, $J$ = 24.0 Hz), 133.06 (d, $J$ = 2.0 Hz), 132.13 (d, $J$ = 3.0 Hz), 131.34, 131.08, 130.97, 130.68, 129.82, 129.72, 128.91, 128.84, 128.80, 128.72, 126.44, 125.96 (d, $J$ = 11.0 Hz), 124.52, 118.33, 117.58, 110.43, 106.44 (d, $J$ = 7.0 Hz), 55.85, 55.23. HRMS: *m/z* [M + Na$^+$] found, 459.1145 ($C_{28}H_{21}O_3PNa$, calcd., 459.1126).

**2,7-Dimethoxy-9-phenyltribenzo[b,e,g]phosphindole 9-oxide (*m*-MOTBPIO-4):** The procedure was analogous to that described for *p*-MOTBPIO. The photoproduct was firstly purified by silica-gel column chromatography. It was further purified by recrystallization with slow solvent evaporation method. Yellow solid, yield 35.8%. $^1$H NMR (400 MHz, $CD_2Cl_2$) $\delta$ (TMS, ppm): 8.65 (d, $J$ = 9.2 Hz, 1H), 8.56 (dd, $J$ = 8.1, 4.6 Hz, 1H), 8.50 (dd, $J$ = 9.2, 1.6 Hz, 1H), 8.34 (d, $J$ = 2.6 Hz, 1H), 7.83–7.77 (m, 1H), 7.76–7.68 (m, 3H), 7.60 (d, $J$ = 1.8 Hz, 1H), 7.51–7.44 (m, 3H), 7.39–7.34 (m, 2H), 7.24 (dd, $J$ = 9.2, 2.7 Hz, 1H), 4.07 (s, 3H), 3.83 (s, 3H). $^{13}$C NMR (100 MHz, $CD_2Cl_2$) $\delta$ (TMS, ppm): 158.74, 143.26 (d, $J$ = 24.0 Hz), 139.35 (d, $J$ = 20.0 Hz), 135.50, 134.44, 133.69 (d, $J$ = 3.0 Hz), 132.56 (d, $J$ = 3.0 Hz), 131.46 (d, $J$ = 10.0 Hz), 131.49 (d, $J$ = 101.0 Hz), 130.29 (d, $J$ = 9.0 Hz), 130.05, 129.96, 129.27 (d, $J$ = 11.0 Hz), 129.21 (d, $J$ = 12.0 Hz), 128.86, 128.70, 128.58, 125.78, 125.67, 125.45, 124.64, 119.08 (d, $J$ = 27.0 Hz), 107.47, 106.68 (d, $J$ = 6.0 Hz), 56.05, 55.78. HRMS: *m/z* [M + H$^+$] found, 437.1313 ($C_{28}H_{22}O_3P$, calcd., 437.1307).

**2.2 X-ray crystallography**

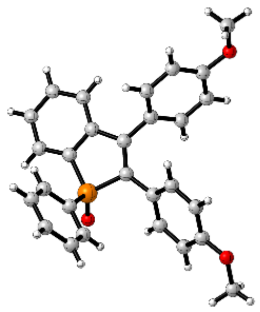

**Crystal data of *p*-MOTPPIO (CCDC 2239437):** $C_{28}H_{23}O_3P$, $M_W$ = 438.43, orthorhombic, Pca2$_1$, $a$ = 22.7203(5) Å, $b$ = 8.7471(2) Å, $c$ = 11.0451(2) Å, $\alpha$ = 90$^o$, $\beta$ = 90$^o$, $\gamma$ = 90$^o$, $V$ = 2195.07(8) Å$^3$, $Z$ = 4, $Dc$ = 1.327 g cm$^{-3}$, $\mu$ = 1.335 mm$^{-1}$ (CuKα, $\lambda$ = 1.54184), $F$(000) = 920,

$T$ = 150(10) K, $2\theta_{max}$ = 67.68° (99.1%), 12723 measured reflections, 3120 independent reflections ($R_{int}$ = 0.024), GOF on $F^2$ = 1.037, $R_1$ = 0.0273, $wR_2$ = 0.065 (all data), $\Delta e$ 0.15 and –0.19 eÅ$^{-3}$.

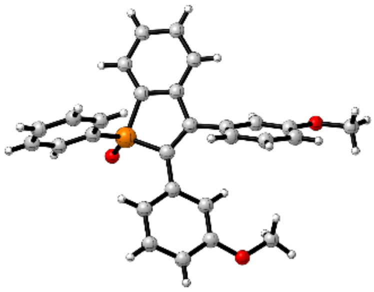

**Crystal data for *m*-MOTPPIO (CCDC 2239443):** $C_{28}H_{23}O_3P$, $M_W$ = 438.43, monoclinic, P$2_1$/n, $a$ = 9.01537(16) Å, $b$ = 24.5128(4) Å, $c$ = 10.20915(18) Å, $\alpha$ = 90°, $\beta$ = 101.9789(17)°, $\gamma$ = 90°, $V$ = 2207(7) Å$^3$, $Z$ = 4, $Dc$ = 1.319 g cm$^{-3}$, $\mu$ = 1.327 mm$^{-1}$ (CuKα, $\lambda$ = 1.54184), $F$(000) = 920, $T$ = 293(2) K, $2\theta_{max}$ = 67.07° (98.4%), 11841 measured reflections, 3886 independent reflections ($R_{int}$ = 0.025), GOF on $F^2$ = 1.039, $R_1$ = 0.0371, $wR_2$ = 0.0859 (all data), $\Delta e$ 0.26 and –0.3 eÅ$^{-3}$.

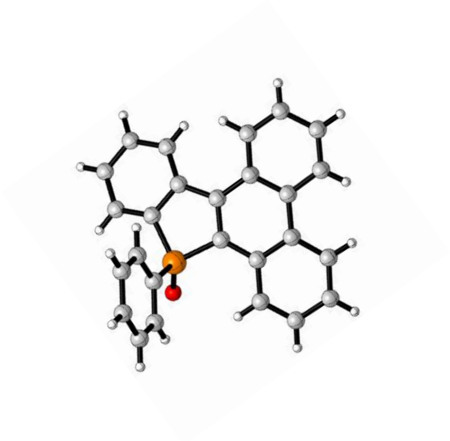

**Crystal data for TBPIO (CCDC 2257996):** $C_{26}H_{17}PO$, $M_W$ = 376.36, monoclinic, c1, $a$ = 17.6986(12) Å, $b$ = 12.9271(13) Å, $c$ = 8.0676(5) Å, $\alpha$ = 90°, $\beta$ = 91.571(6)°, $\gamma$ = 90°, $V$ = 1845.1(3) Å$^3$, $Z$ = 4, $Dc$ = 1.355 g cm$^{-3}$, $\mu$ = 1.416 mm$^{-1}$ (CuKα, $\lambda$ = 1.54184), $F$(000) = 784, $T$ = 153.7(8) K, $2\theta_{max}$ = 67.684° (98.9%), 4535 measured reflections, 2379 independent reflections ($R_{int}$ = 0.0527), GOF on $F^2$ = 1.082, $R_1$ = 0.0708, $wR_2$ = 0.1775 (all data), $\Delta e$ 0.886 and –0.693 eÅ$^{-3}$.

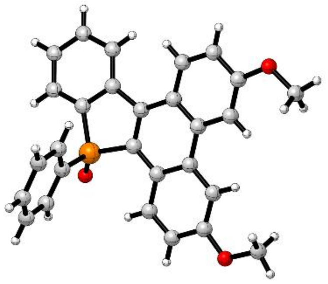

**Crystal data for *p*-MOTBPIO (CCDC 2239442):** $C_{28}H_{21}PO_3$, $M_W$ = 436.42, monoclinic, $P2_1/c$, $a$ = 8.248(2) Å, $b$ = 21.038(4) Å, $c$ = 12.6398(2) Å, $\alpha$ = 90°, $\beta$ = 105.93(2)°, $\gamma$ = 90°, $V$ = 2109.05(8) Å$^3$, $Z$ = 4, $Dc$ = 1.374 g cm$^{-3}$, $\mu$ = 1.389 mm$^{-1}$ (CuKα, $\lambda$ = 1.54184), $F$(000) = 912, $T$ = 293(2) K, $2\theta_{max}$ = 67.68° (99.8%), 10068 measured reflections, 4125 independent reflections ($R_{int}$ = 0.0131), GOF on $F^2$ = 1.077, $R_1$ = 0.0367, $wR_2$ = 0.0954 (all data), $\Delta e$ 0.23 and –0.55 eÅ$^{-3}$.

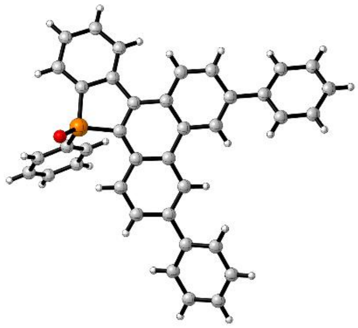

**Crystal data for *p*-PhTBPIO (CCDC 2239447):** $C_{38}H_{25}OP$, $M_W$ = 528.55, monoclinic, $P2_1/n$, $a$ = 12.6529(2) Å, $b$ = 15.6034(2) Å, $c$ = 13.8134(2) Å, $\alpha$ = 90°, $\beta$ = 98.3440(10)°, $\gamma$ = 90°, $V$ = 2699.67(7) Å$^3$, $Z$ = 4, $Dc$ = 1.300 g cm$^{-3}$, $\mu$ = 1.129 mm$^{-1}$ (CuKα, $\lambda$ = 1.54184), $F$(000) = 1104, $T$ = 293(2) K, $2\theta_{max}$ = 67.68° (99.3%), 17883 measured reflections, 5294 independent reflections ($R_{int}$ = 0.0328), GOF on $F^2$ = 1.031, $R_1$ = 0.0378, $wR_2$ = 0.0973 (all data), $\Delta e$ 0.27 and –0.41 eÅ$^{-3}$.

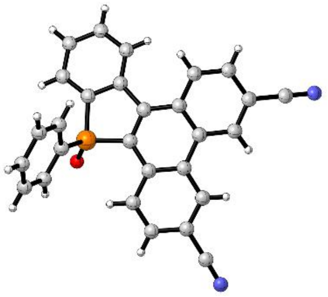

**Crystal data for *p*-CNTPPIO (CCDC 2239441):** $C_{28}H_{15}N_2OP$, $M_W$ = 411.77, triclinic, P-1, $a$ = 8.7794(3) Å, $b$ = 9.9425(3) Å, $c$ = 12.4182(3) Å, $\alpha$ = 68.803(3)°, $\beta$ = 78.720(2)°, $\gamma$ = 82.079(3)°, $V$ = 988.44(6) Å$^3$, $Z$ = 2, $Dc$ = 1.384 g cm$^{-3}$, $\mu$ = 1.426 mm$^{-1}$ (CuKα, $\lambda$ = 1.54184), $F$(000) = 411, $T$ = 293(2) K, $2\theta_{max}$ = 67.68° (98.1%), 8259 measured reflections, 3804 independent reflections ($R_{int}$ = 0.0173), GOF on $F^2$ = 1.095, $R_1$ = 0.0391, $wR_2$ = 0.1050 (all data), Δ$e$ 0.31 and –0.61 eÅ$^{-3}$.

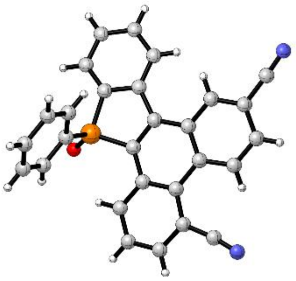

**Crystal data for *m*-CNTBPIO-1 (CCDC 2239444):** $C_{28}H_{15}N_2OP$, $M_W$ = 426.39, triclinic, P-1, $a$ = 8.2254(3) Å, $b$ = 11.6409(3) Å, $c$ = 12.1555(3) Å, $\alpha$ = 88.045(2)°, $\beta$ = 88.158(2)°, $\gamma$ = 88.241(2)°, $V$ = 1162.11(6) Å$^3$, $Z$ = 2, $Dc$ = 1.219 g cm$^{-3}$, $\mu$ = 1.215 mm$^{-1}$ (CuKα, $\lambda$ = 1.54184), $F$(000) = 440, $T$ = 293(2) K, $2\theta_{max}$ = 67.68° (98.2%), 12503 measured reflections, 4475 independent reflections ($R_{int}$ = 0.0179), GOF on $F^2$ = 1.122, $R_1$ = 0.0725, $wR_2$ = 0.2085 (all data), Δ$e$ 1.33 and –0.93 eÅ$^{-3}$.

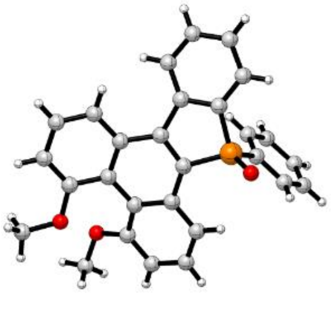

**Crystal data for *m*-MOTBPIO-1 (CCDC 2259430):** $C_{28}H_{21}O_3P$, $M_W$ = 436.42, triclinic, P-1, $a$ = 7.9627(3) Å, $b$ = 10.2123(3) Å, $c$ = 26.0920(6) Å, $\alpha$ = 88.559(2)°, $\beta$ = 82.803(3)°, $\gamma$ =83.855(3)°, $V$ = 2092.78(11) Å$^3$, $Z$ = 4, $Dc$ = 1.385 g cm$^{-3}$, $\mu$ = 1.4 mm$^{-1}$ (CuKα, $\lambda$ = 1.54184), $F$(000) = 912, $T$ = 150(10) K, $2\theta_{max}$ = 67.079° (97.9%), 19902 measured reflections, 7335 independent reflections ($R_{int}$ = 0.0912), GOF on $F^2$ = 1.109, $R_1$ = 0.1065, $wR_2$ = 0.3323 (all data), Δ$e$ 1.455 and –0.464 eÅ$^{-3}$.

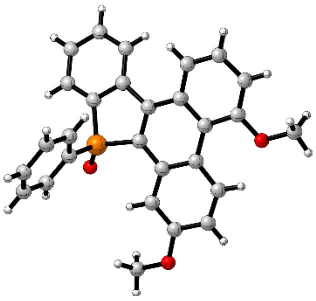

**Crystal data for *m*-MOTBPIO-2 (CCDC 2239449):** $C_{28}H_{21}O_3P$, $M_W$ = 436.42, monoclinic, P2$_1$/n, $a$ = 11.2403(2) Å, $b$ = 11.8812(3) Å, $c$ = 16.3332(3) Å, $\alpha$ = 90°, $\beta$ = 105.44(2)°, $\gamma$ = 90°, $V$ = 2102.55(8) Å$^3$, $Z$ = 4, $Dc$ = 1.379 g cm$^{-3}$, $\mu$ = 1.393 mm$^{-1}$ (CuKα, $\lambda$ = 1.54184), $F$(000) = 912, $T$ = 293(2) K, $2\theta_{max}$ = 67.68° (100%), 13659 measured reflections, 4174 independent reflections ($R_{int}$ = 0.0188), GOF on $F^2$ = 1.055, $R_1$ = 0.0357, $wR_2$ = 0.0859 (all data), Δ$e$ 0.3 and –0.37 eÅ$^{-3}$.

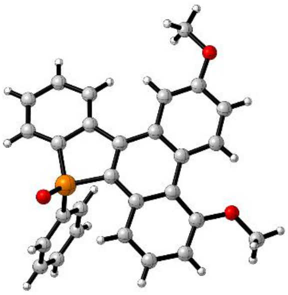

**Crystal data for *m*-MOTBPIO-3 (CCDC 2255373):** $C_{28}H_{21}O_3P$, $M_W$ = 436.42, triclinic, P-1, $a$ = 9.3112(3) Å, $b$ = 10.5595(3) Å, $c$ = 11.6291(3) Å, $\alpha$ = 87.606(2)°, $\beta$ = 88.772(2)°, $\gamma$ = 67.590(3)°, $V$ = 1056.10(6) Å$^3$, $Z$ = 2, $Dc$ = 1.372 g cm$^{-3}$, $\mu$ = 1.387 mm$^{-1}$ (CuKα, $\lambda$ = 1.54184), $F$(000) = 456, $T$ = 150.00(10) K, $2\theta_{max}$ = 67.072° (98.1%), 8970 measured reflections, 3689

independent reflections ($R_{int}$ = 0.0138), GOF on $F^2$ = 1.053, $R_1$ = 0.0341, $wR_2$ = 0.0889 (all data), Δ$e$ 0.295 and –0.425 eÅ$^{-3}$.

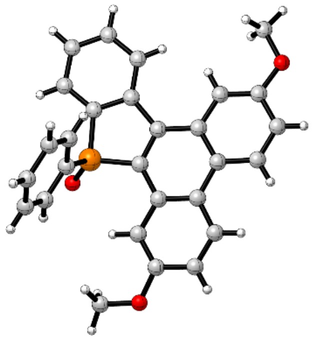

**Crystal data for *m*-MOTBPIO-4 (CCDC 2239445):** $C_{28}H_{21}O_3P$, $M_W$ = 436.42, triclinic, P-1, $a$ = 7.674(3) Å, $b$ = 12.3215(4) Å, $c$ = 12.4534(3) Å, $\alpha$ = 72.72(3)$^o$, $\beta$ = 75.021(3)$^o$, $\gamma$ = 71.969(3)$^o$, $V$ = 1051.03(7) Å$^3$, $Z$ = 2, $Dc$ = 1.379 g cm$^{-3}$, $\mu$ = 1.393 mm$^{-1}$ (CuKα, $\lambda$ = 1.54184), $F$(000) = 456, $T$ = 293(2) K, 2$\theta_{max}$ = 67.68$^o$ (98.3%), 10761 measured reflections, 4064 independent reflections ($R_{int}$ = 0.0186), GOF on $F^2$ = 1.058, $R_1$ = 0.0352, $wR_2$ = 0.0925 (all data), Δ$e$ 0.22 and –0.42 eÅ$^{-3}$.

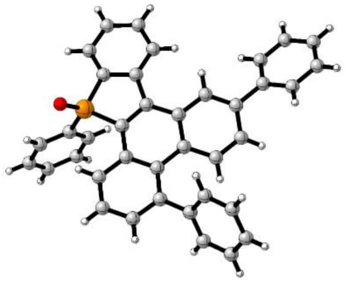

**Crystal data for *m*-PhTBPIO-1 (CCDC 2239446):** $C_{38}H_{25}OP$, $M_W$ = 536.9, monoclinic, I2/a, $a$ = 26.014(3) Å, $b$ = 8.907(8) Å, $c$ = 23.7243(17) Å, $\alpha$ = 90$^o$, $\beta$ = 105.194(9)$^o$, $\gamma$ = 90$^o$, $V$ = 5305(9) Å$^3$, $Z$ = 8, $Dc$ = 1.344 g cm$^{-3}$, $\mu$ = 1.578 mm$^{-1}$ (CuKα, $\lambda$ = 1.54184), $F$(000) = 2239, $T$ = 293(2) K, 2$\theta_{max}$ = 67.68$^o$ (98.8%), 15001 measured reflections, 5199 independent reflections ($R_{int}$ = 0.0424), GOF on $F^2$ = 0.942, $R_1$ = 0.0894, $wR_2$ = 0.2475 (all data), Δ$e$ 1.16 and –0.54 eÅ$^{-3}$.

**2.3 Theoretical calculation**

The ground-state ($S_0$) and excited-state ($S_1$, $T_1$, and $T_2$) geometries were evaluated by the density function theory (DFT) and time-dependent density function theory (TD-DFT) method at the level of M06-2X/6-31G (d), performed using Gaussian 16 package, Revision D.01. The electron cloud analysis was performed by Multiwfn 3.6[6] and VMD 1.9.3.[7] The spin-orbit coupling (SOC) values and the transition states (TS) were calculated by ORCA. The combined QM/MM method with a two-layer ONIOM approach and electronic embedding scheme was used to simulate the aggregation effect on the central molecule, where one central molecule was defined as the QM region and the surrounding molecules were defined as the MM region. The ground-state and excited-state calculations of the isolated-state molecule in the dimethylsulfoxide (DMSO) and aggregated-state molecule in the QM region were performed using DFT and the TD-DFT method, respectively. Geometry comparisons and root-mean-square displacement (RMSD) values between $S_0$ and $S_1$structures were performed by VMD 1.9.3. The transition states (TS) search was done on the quantum mechanics package ORCA 4.1.

**2.4 EPR spectroscopy and HRMS**

DMPO was employed as spin-trapping agent to detect the generation of radical. The DMSO solution containing 25 mM DMPO was mixed separately with *p*-MOTPPIO, *p*-PhTPPIO, *p*-CNTPPIO, *m*-MOTPPIO, *m*-PhTPPIO or *m*-CNTPPIO. The EPR spectra were recorded after the mixture was irradiated with 365 nm UV light. The EPR spectra of *p*-MOTPPIO solid without DMPO was also measured under continuous UV irradiation. EPR spectra of spin were monitored in a range of 3000–4000 G.

The HRMS for DMPO adducts of *p*-MOTPPIO, *p*-PhTPPIO, *p*-CNTPPIO, *m*-MOTPPIO, *m*-PhTPPIO or *m*-CNTPPIO were measured in tetrahydrofuran (THF) solution with addition of 8 *equiv.* of DMPO after 365 nm UV irradiation.

**2.5 ROS generation measurement in aqueous media**

*2.5.1 General ROS detection*

The general ROS generation measurements were conducted using 2,7-dichlorodihydrofluorescein (DCFH) as the indicator, which was converted from DCFH-DA (0.5 mL, 1 mM in ethanol) reacting with an aqueous solution of NaOH (2 mL, 1.0 mM) for 30 min at a room temperature. The hydrolysate was then neutralized with 7.5 mL PBS buffer solution to get the stock solution with a concentration of 50 μM. PBS buffer solution containing 10 μM DCFH was added with 10 μM *p*-MOTPPIO or *p*-MOTBPIO (stock solution: 1 mM in DMSO). The irradiation input power was adjusted to 10 mW $cm^{-2}$ by changing the distance between the lamp and the solution. The fluorescence signal of the indicator was monitored in a range of 510–560 nm with the excitation wavelength of 504 nm after the solution was irradiated. The fluorescence intensity change at 522 nm was recorded to indicate the ROS generation rate.

*2.5.2 $O_2^{\bullet-}$ detection*

The $O_2^{\bullet-}$ generation measurements were conducted using DHR123 as the indicator. PBS buffer solution containing 10 μM DHR123 (stock solution: 5 mM in DMF) was added wih 10 μM *p*-MOTPPIO or *p*-MOTBPIO (stock solution: 1 mM in DMSO). The fluorescence signal of the indicator was monitored in a range of 500–650 nm with the excitation wavelength of 480 nm after the solution was irradiated by white light irradiation of 10 mW $cm^{-2}$. The fluorescence intensity at 520 nm was recorded to indicate the $O_2^{\bullet-}$ generation rate.

*2.5.3 $^1O_2$ detection*

The $^1O_2$ generation measurements were conducted using ABDA as the indicator. PBS buffer solution containing 40 μM ABDA (stock solution: 10 mM in DMSO) was added with 10 μM *p*-MOTPPIO or *p*-MOTBPIO or RB (stock solution: 1 mM in (DMSO). The absorption spectra of the indicator were monitored in a range of 330–450 nm with the excitation wavelength of 480 nm after the solution was irradiated by white light irradiation of 20 mW $cm^{-2}$. The absorbance decline relative to the initial value at 380 nm was recorded to indicate the decomposition rates of ABDA ($^1O_2$ generation rate).

**2.6 Cell study**

*2.6.1 Cell culture*

HeLa cells were cultured in the DMEM supplemented with 10% FBS and 1% penicillin-streptomycin at 37 $^oC$ in a humidified incubator with 5% $CO_2$.

*2.6.2 Oleic acid induced accumulation of LDs*

Hela cells were pretreated with 100 μg $mL^{-1}$ OA for 6–12 h under incubator conditions.

*2.6.3 Dark toxicity assays*

HeLa cells were seeded in 96-well plates at a density of $1 \times 10^5$ cells/mL. After 24 h of culture, different concentrations (0 to 30 μM) of *p*-MOTPPIO and *p*-MOTBPIO were added and incubated at 37 $^oC$ for another 24 h in dark. The sample and control wells were removed and added with freshly prepared 100 μL MTT solution (0.5 mg $mL^{-1}$). After incubation at 37 $^oC$ for 2–4 h, the MTT solution was removed and 100 μL DMSO was then added into each well and the plate was shaken for 3 min at room temperature to dissolve all the precipitates formed. The absorbance of the sample and control wells at 565 nm was then measured by a microplate reader. Cell viability was then calculated by the ratio of the absorbance of sample wells to control cells.

*2.6.4 Cell imaging*

HeLa cells were seeded in confocal imaging dish at a density of 5 × $10^4$ cells per dish and cultured for 24 h to allow cells to adhere adequately. Then the medium was replaced by a fresh medium containing 5 μM *p*-MOTPPIO or *p*-MOTBPIO. After incubated for 0.5/1/4/8 h at 37 $^{o}$C or 4 $^{o}$C, the cells were imaged by CLSM in the channel mode as well as lambda mode at excitation wavelength of 405 nm.

*2.6.5 Confocal co-localization*

HeLa cells were grown in a confocal imaging dish at 37 $^{o}$C. HeLa cells were firstly incubated with 1 mL medium containing 5 μM *p*-MOTPPIO or *p*-MOTBPIO at 37 $^{o}$C for 1 h. After rinsed with PBS for three times and then stained with medium containing 1 μM commercial dyes ER-Tracker Red (1 μM, stock solution: 1 mM in DMSO), Nile red (1 μM, stock solution: 1 mM in DMSO) at 37 $^{o}$C for 30 min, the cells were imaged using CLSM. For *p*-MOTPPIO and *p*-MOTBPIO, the excitation wavelength was 405 nm and the collected emission range was 430–550 nm; for ER-Tracker Red the excitation wavelength was 543 nm, and the collected emission range was 590–650 nm; and for Nile Red, the excitation wavelength was 488 nm, and the collected emission range was 540–650 nm.

*2.6.7 PDT in vitro*

HeLa cells were seeded in 96-well plates at a density of 1 × $10^5$ cells/mL. After 24 h of culture, different concentrations (0 to 30 μM) of *p*-MOTPPIO or *p*-MOTBPIO were added and incubated at 37 $^{o}$C for 1 h, followed by irradiation (white light: 20 mW $cm^{-2}$, 10 min or 365 nm UV: 10 mW $cm^{-2}$, 1 min). The cells were further incubated at 37 $^{o}$C to 24 h. MTT assay was conducted as described in 2.6.3.

*2.6.8 Detection of extracellular ROS generation*

HeLa cells were grown in a confocal imaging dish at 37 $^{o}$C. After incubation with medium containing 5 μM *p*-MOTBPIO (stock solution: 1 mM in DMSO) or pure DMSO for 1 h and treatment with medium containing 10 μM DCFH-DA (stock solution: 1 mM in ethanol) for 30

min at 37 $^{o}C$, the cells were imaged by CLSM at excitation wavelength of 488 nm with continuous irradiation of 405 nm laser of 5% power in the designated area. The signal intensity was recorded at every 20 s and measured using ZEN 2012 (blue edition) software. The collected emission range was 490–720 nm.

2*.6.9 Detection of extracellular lipid peroxides*

HeLa cells were grown in a confocal imaging dish at 37 $^{o}C$. After incubation with medium containing 5 μM *p*-MOTBPIO (stock solution: 1 mM in DMSO) or pure DMSO for 1 h and treatment with medium containing 10 μM Liperfluo (stock solution: 1 mM in DMSO) for 30 min at 37 $^{o}C$, the cells were imaged by CLSM at excitation wavelength of 488 nm with continuous irradiation of 405 nm laser of 8% power in the designated area. The signal intensity was recorded at every 20 s and measured using ZEN 2012 (blue edition) software. The collected emission range was 500–650 nm.

*2.6.10 Detection of extracellular lipid peroxidation MDA*

HeLa cells were grown in a large petri dish at 37 $^{o}C$. After incubation with medium containing 5 μM *p*-MOTBPIO (stock solution: 1 mM in DMSO) or pure DMSO for 1 h. With the exposure to UV irradiation of 20 mW $cm^{-2}$ for 0/5/10 min. The supernatants were collected and the level of secreted MDA was quantitatively determined by Lipid Peroxidation MDA Assay Kit according to the manufacturer's instruction.

*2.6.11 Inhibition of intracellular lipid peroxide production*

HeLa cells were grown in a confocal imaging dish at 37 $^{o}C$. After incubation with medium containing 5 μM *p*-MOTBPIO (stock solution: 1 mM in DMSO) for 1 h and treatment with medium containing 0.5 mM Vc (stock solution: 500 mM in $H_2O$) or 2 μM Fer-1 (stock solution: 1 mM in $H_2O$) for 30 min at 37 $^{o}C$, the cells were imaged by CLSM at excitation wavelength of 405 nm with continuous irradiation of 405 nm laser of 2% power in the designated area. The signal intensity was recorded at every 1 s and measured using ZEN 2012 (blue edition) software.

## 3.0 Supplementary Figures and Tables

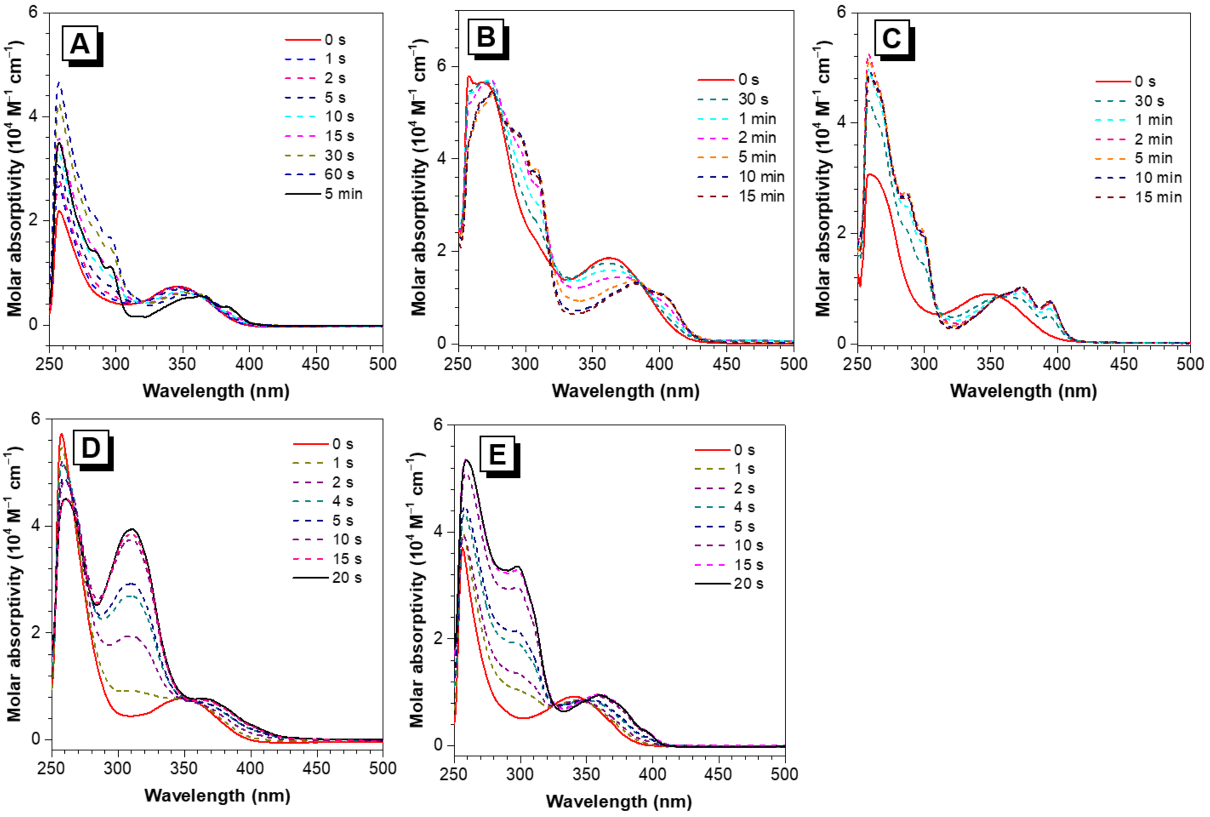


**Figure S1.** UV–vis absorption spectral changes of (A) TPPIO (10 μM), (B) *p*-PhTPPIO (10 μM), (C) *p*-CNTPPIO (10 μM), (D) *m*-PhTPPIO (10 μM), and (E) *m*-CNTPPIO (10 μM) in DMSO upon 365 nm UV light irradiation for different time.

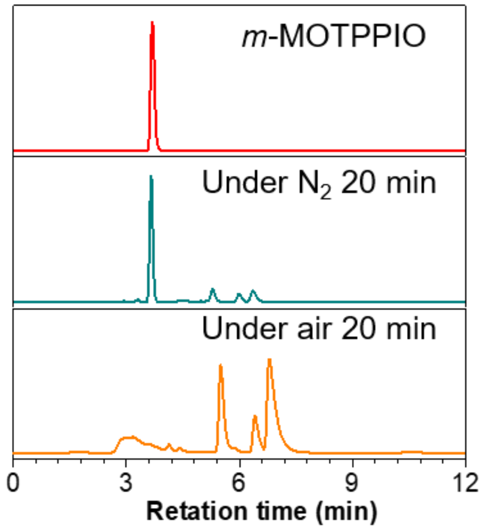


**Figure S2**. HPLC spectral changes of *m*-MOTPPIO upon 365 nm UV light irradiation in MeCN under air or $N_2$ (the flask containing reaction solution is frozen with liquid nitrogen and then subjucted to three vacuum extraction and $N_2$ filling) for 20 min (measured at 254 nm).

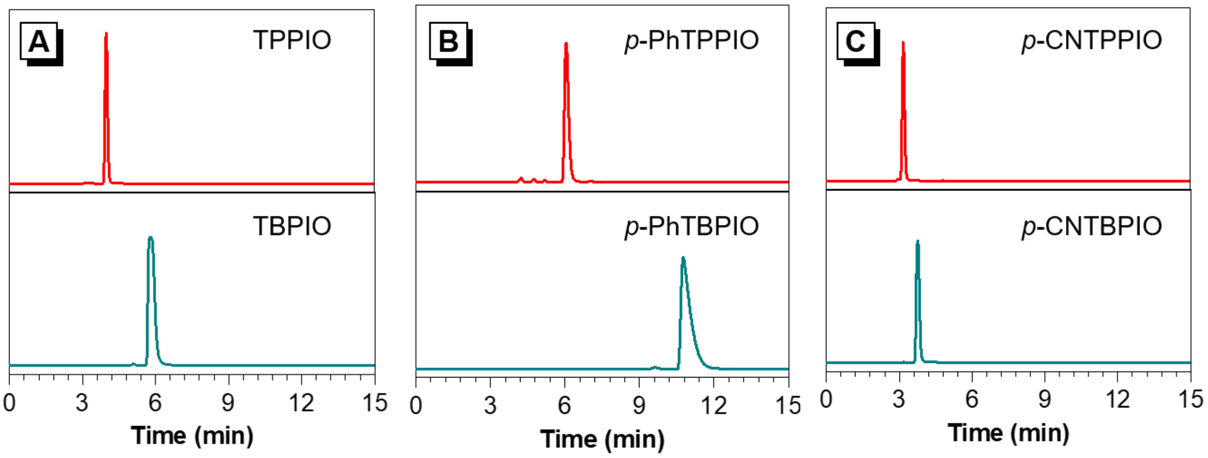


**Figure S3.** HPLC spectral of (A)TPPIO, TBPIO (B) *p*-PhTPPIO, *p*-PhTBPIO and (C) *p*-CNTPPIO, *p*-CNTBPIO in MeCN (measured at 254 nm).

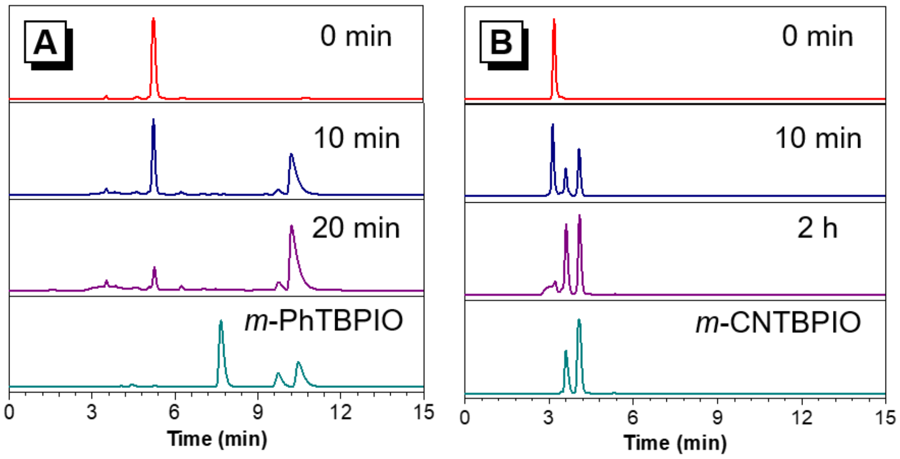


**Figure S4**. HPLC spectral changes of (A) *m*-PhTPPIO, and (B) *m*-CNTPPIO upon 365 nm UV light irradiation in MeCN for different time (measured at 254 nm).

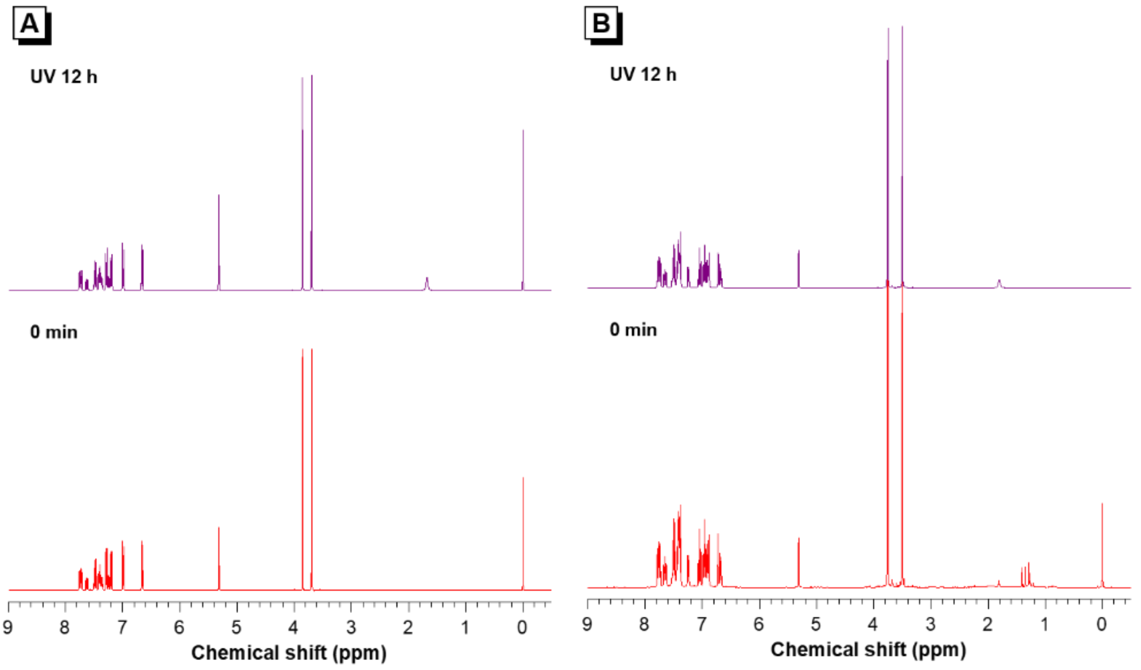


**Figure S5**. $^{1}$H NMR spectra of (A) *p*-MOTPPIO (B) *m*-MOTPPIO measured in $CD_2Cl_2$ after the solid powder with and without 365 nm UV light irradiation.

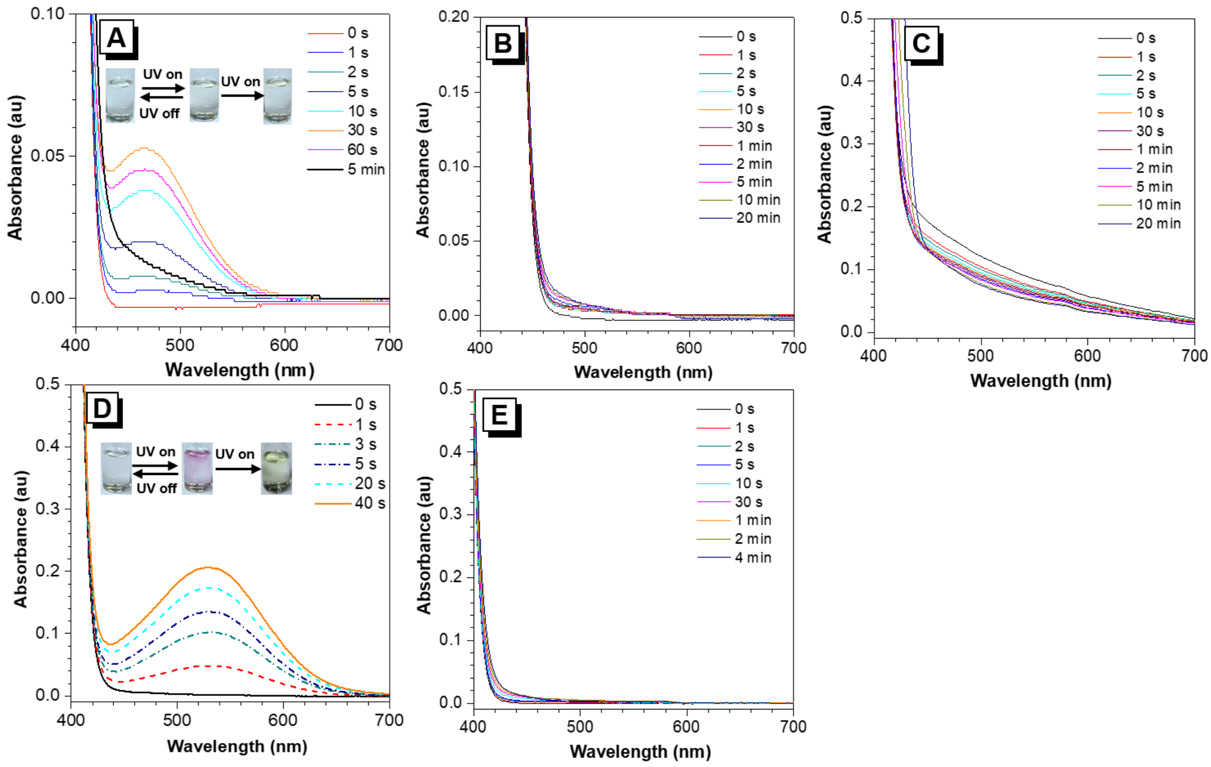


**Figure S6**. UV–vis absorption spectral changes of (A) TPPIO, (B) *p*-PhTPPIO (5 mM), (C) *p*-CNTPPIO (5 mM), (D) *m*-PhTPPIO (5 mM), and (E) *m*-CNTPPIO (5 mM) in DCM upon 365 nm UV light irradiation for different time.

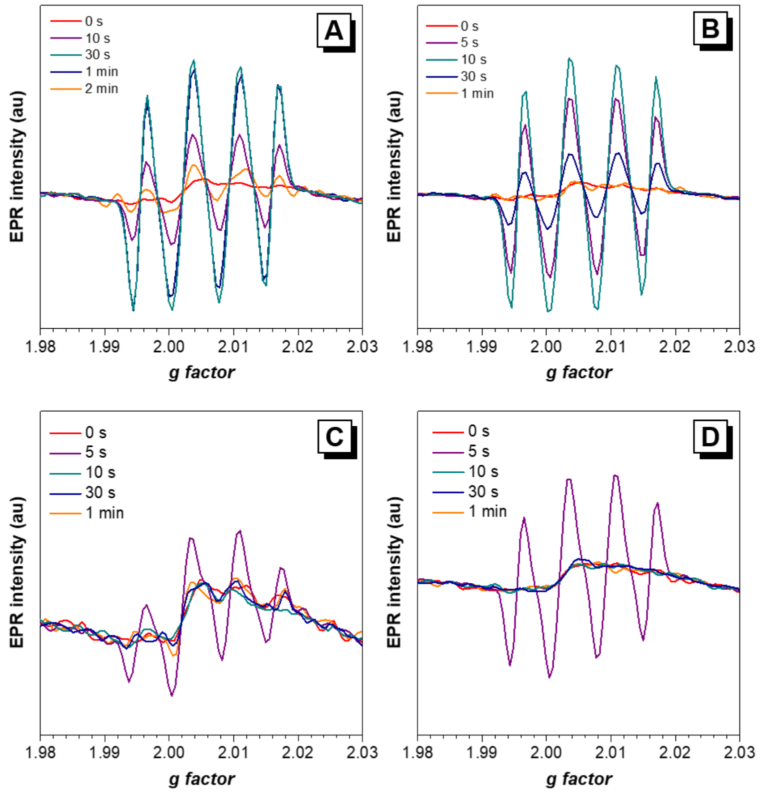


**Figure S7**. EPR spectral changes of (A) *p*-PhTPPIO (1 mM), (B) *p*-CNTPPIO (1 mM), (C) *m*-PhTPPIO (1 mM), and (D) *m*-CNTPPIO (1 mM) in DMSO solution with the addition of DMPO (25 mM) under 365 nm UV light irradiation for different time.

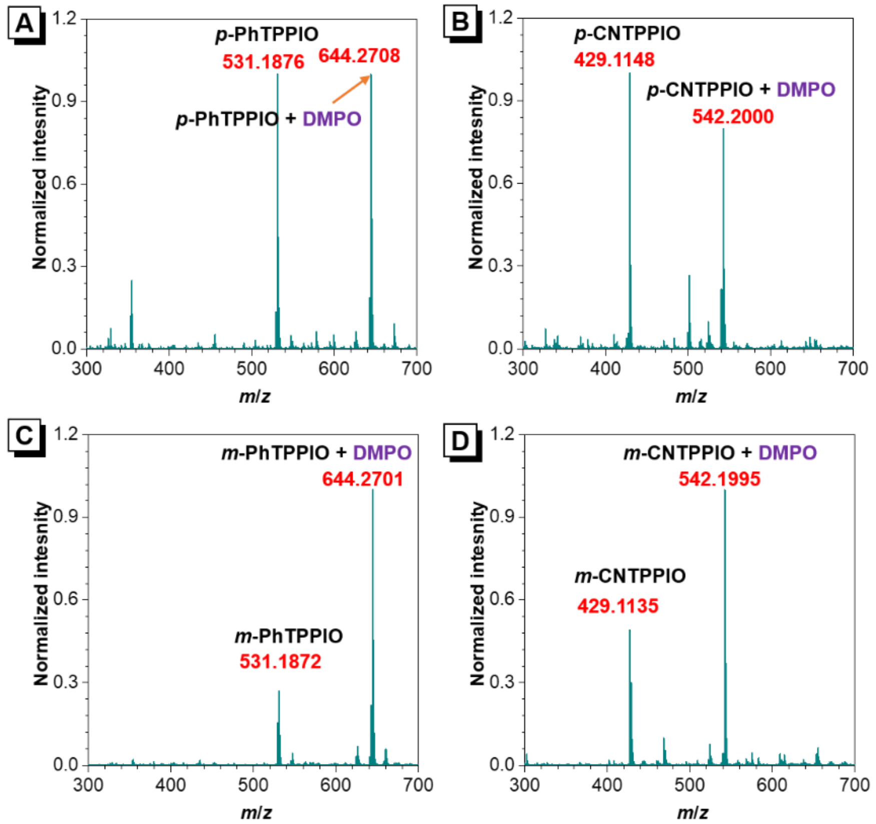


**Figure S8.** HRMS spectra of (A) *p*-PhTPPIO (calcd., 531.1878), (B) *p*-CNTPPIO (calcd., 429.1157), (C) *m*-PhTPPIO (calcd., 531.1878), and (D) *m*-CNTPPIO (calcd., 429.1157) in

THF solution with the addition of 8 *equiv.* of DMPO after 365 nm UV irradiation (the calculated MW for DMPO adducts of Ph- and CN-substituted molecules are 644.2719 and 542.1998, respectively).

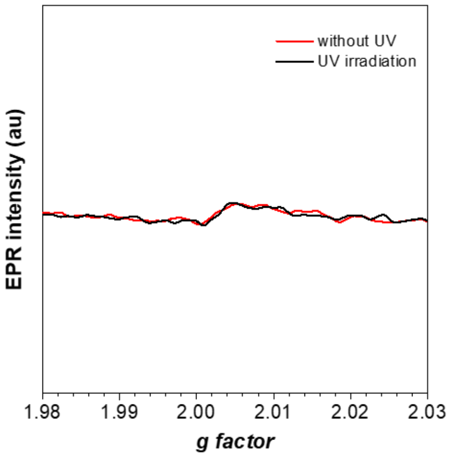


**Figure S9**. EPR spectra of *p*-MOTPPIO solid powder with and without 365 nm UV light irradiation (measured in powder form).

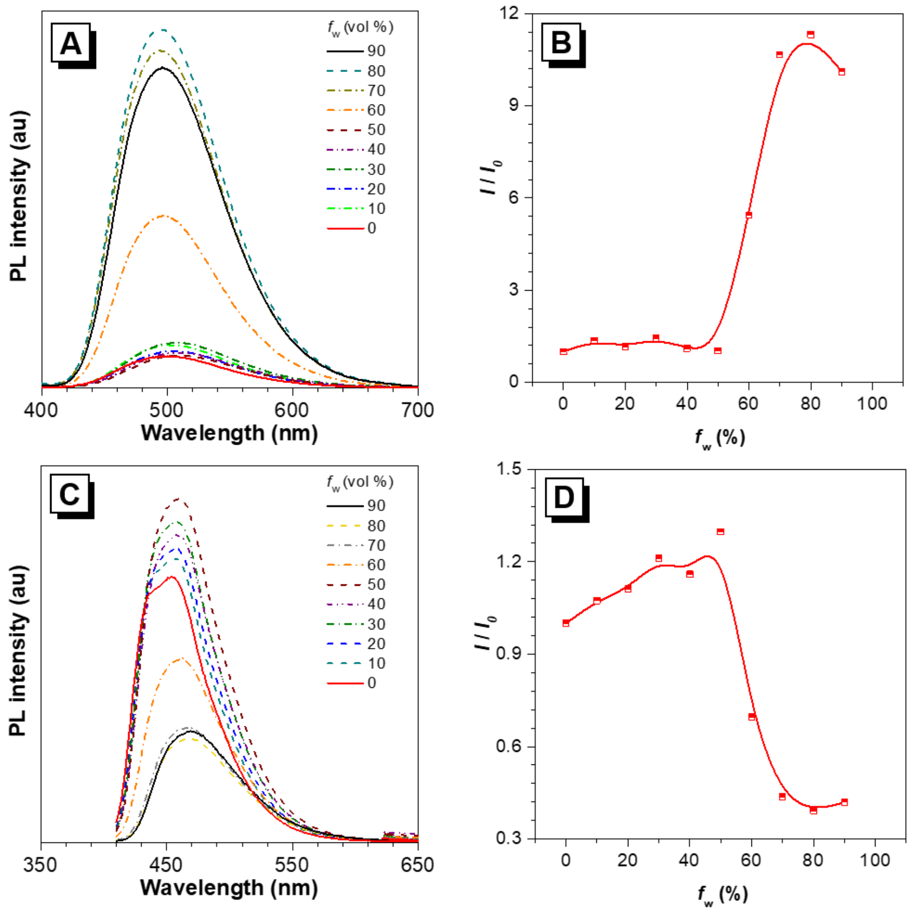


**Figure S10**. PL spectra and plots of $I/I_0$ *vs.* $f_w$ for (A, B) *p*-PhTPPIO and (C, D) *p*-PhTBPIO in $H_2O$/DMSO mixtures with different $H_2O$ fractions. $I_0$ is the PL intensity of the sample at $f_w$ = 0 vol%.

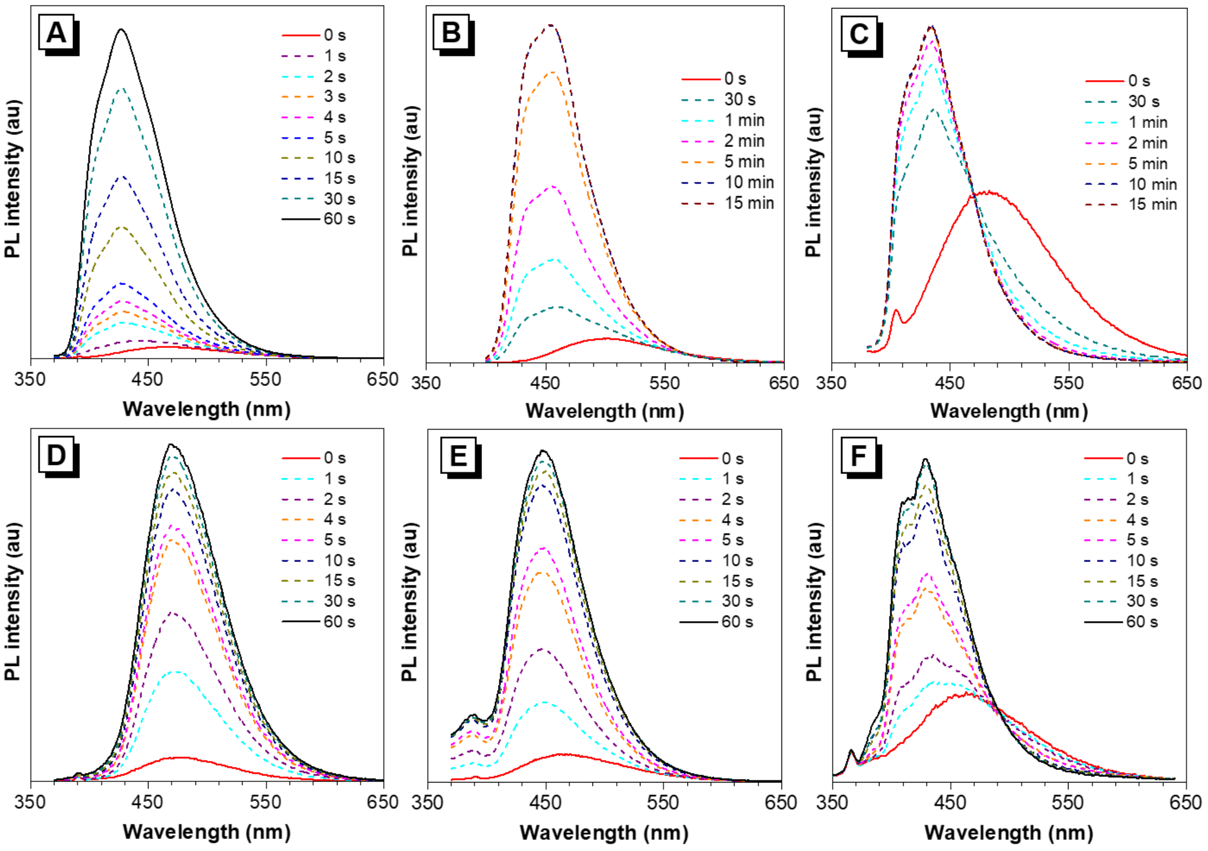


**Figure S11.** PL spectral changes of (A) TPPIO (10 µM), (B) *p*-PhTPPIO (10 µM), (C) *p*-CNTPPIO (10 µM), (D) *m*-MOTPPIO (10 µM), (E) *m*-PhTPPIO (10 µM), and (F) *m*-CNTPPIO (10 µM) in DMSO upon 365 nm UV light irradiation for different time.

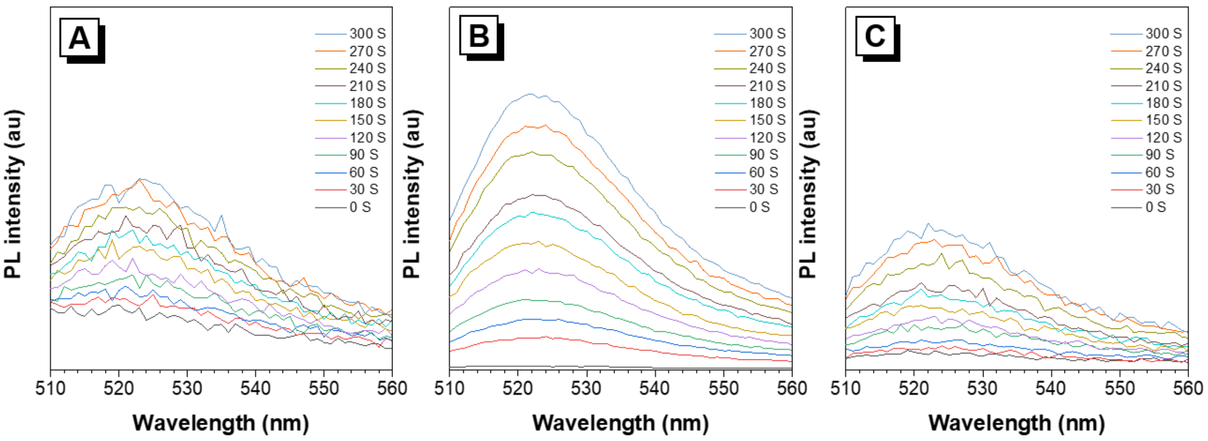


**Figure S12.** PL intensity of DCFH with (A) *p*-MOTPPIO (10 µM), (B) *p*-MOTBPIO (10 µM), and (C) control in $H_2O$ with 1 vol% DMSO under white light irradiation (10 mW $cm^{-2}$).

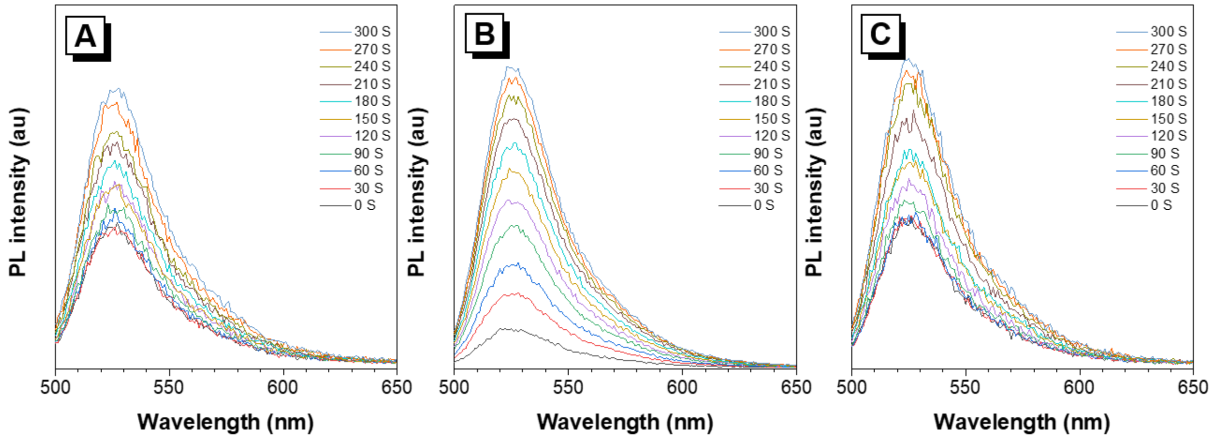


**Figure S13.** PL intensity of DHR123 with (A) *p*-MOTPPIO (10 μM), (B) *p*-MOTBPIO (10 μM), and (C) control in $H_2O$ with 1 vol% DMSO under white light irradiation (10 mW cm$^{-2}$).

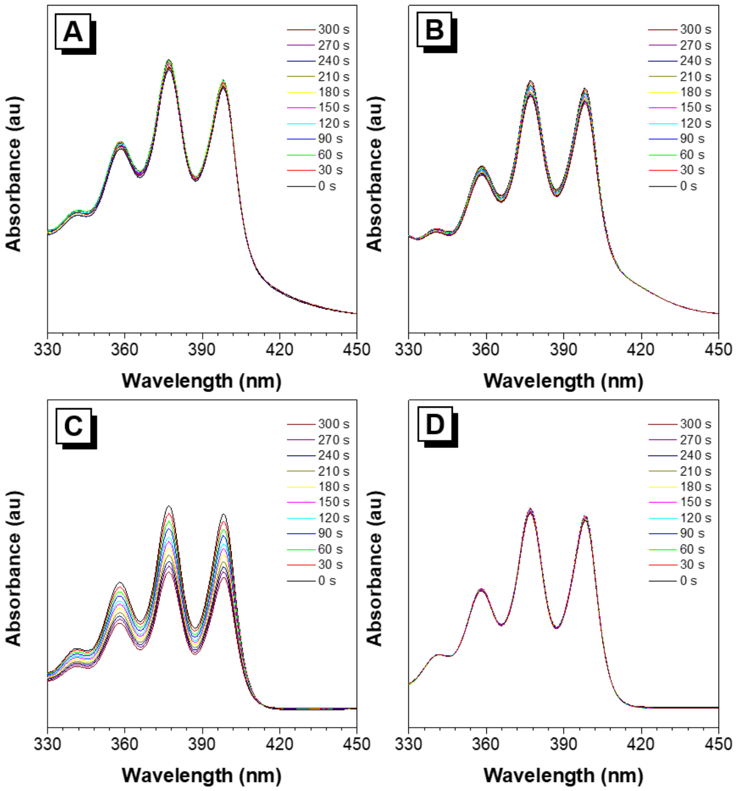


**Figure S14**. Photodegradation of ABDA with (A) *p*-MOTPPIO (10 μM), (B) *p*-MOTBPIO (10 μM), (C) RB (10 μM) and (D) Control in $H_2O$ with 1 vol% DMSO under white light irradiation (10 mW cm$^{-2}$).

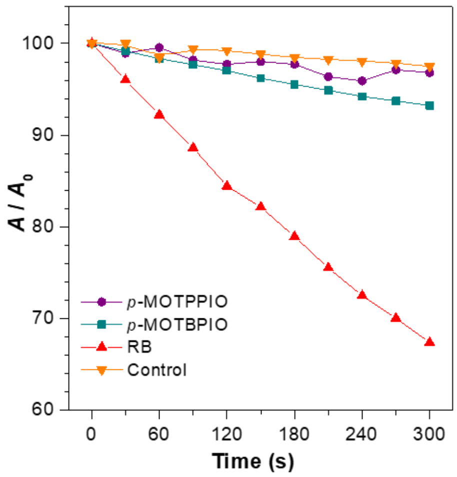


**Figure S15.** Plots of $A/A_0$ *vs.* irradiation time for the $^1O_2$ generation of *p*-MOTPPIO (10 μM), *p*-MOTBPIO (10 μM), RB (10 μM) and control in $H_2O$ with 1 vol% DMSO using ABDA as indicator under white light irradiation (10 mW $cm^{-2}$); $A_0$ is the absorbance of ABDA before irradiation.

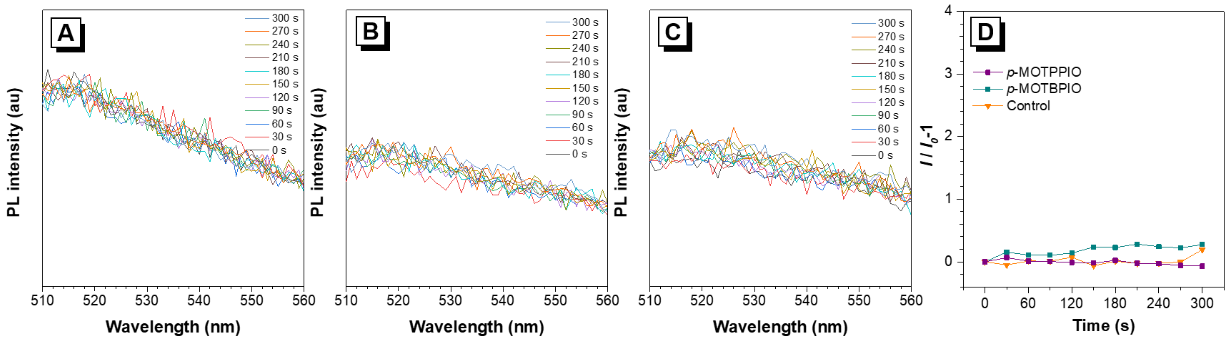


**Figure S16.** PL intensity of HPF with (A) *p*-MOTPPIO (10 μM), (B) *p*-MOTBPIO (10 μM), and (C) control in $H_2O$ with 1 vol% DMSO under white light irradiation (10 mW $cm^{-2}$). And (D) plots of $I/I_0 - 1$ *vs.* irradiation time for the $OH^{\bullet}$ generation of *p*-MOTPPIO, *p*-MOTBPIO, and control.

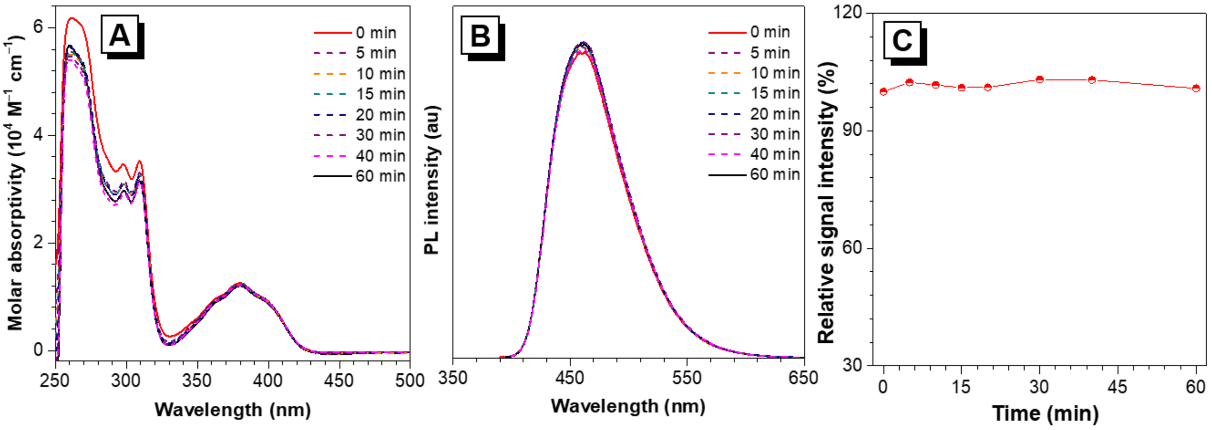


**Figure S17**. (A) UV–vis absorption (B) PL spectral changes of *p*-MOTBPIO in DMSO, and (C) the relative signal intensity of the emission under 365 nm UV irradiation for different time.

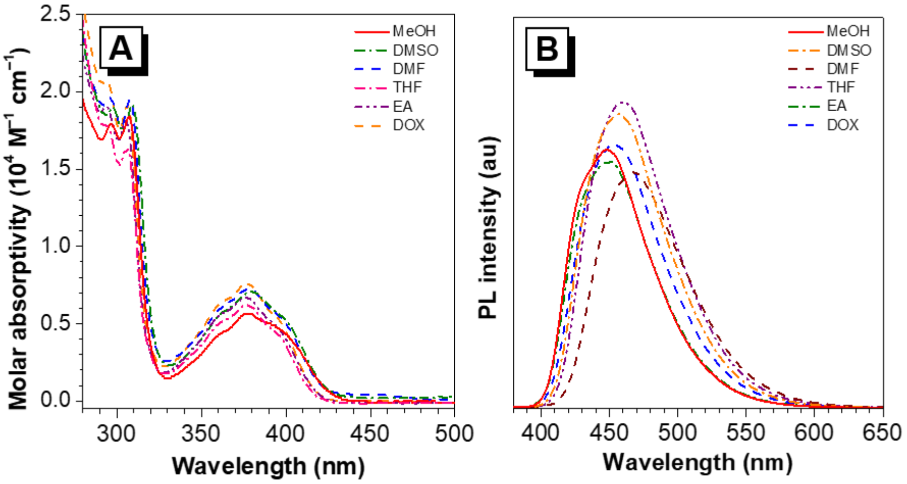


**Figure S18.** (A) UV–vis absorption (B) PL spectral changes of *p*-MOTBPIO in different solutions with various polarity.

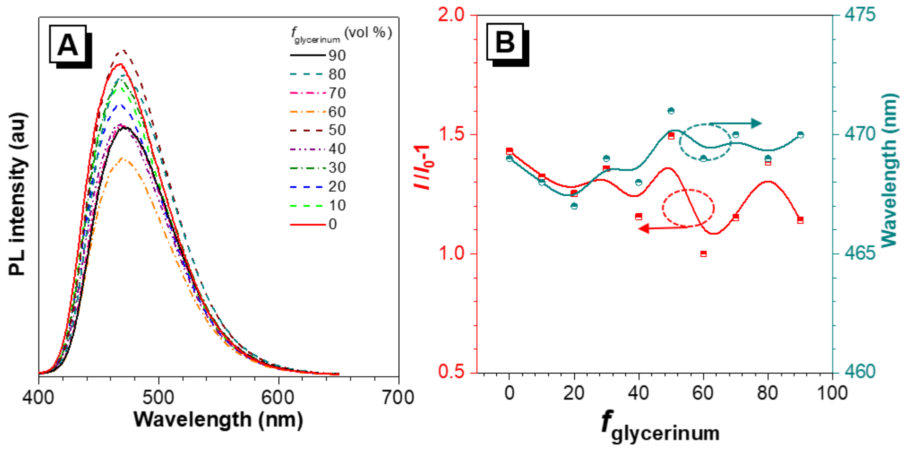


**Figure S19**. (A) PL spectra and (B) plots of $I/I_0$ *vs.* $f_{glycerinum}$ for *p*-MOTBPIO in glycerinum/MeOH mixtures with different glycerinum fractions. $I_0$ is the PL intensity of the sample at $f_{glycerinum}$ = 0 vol%.

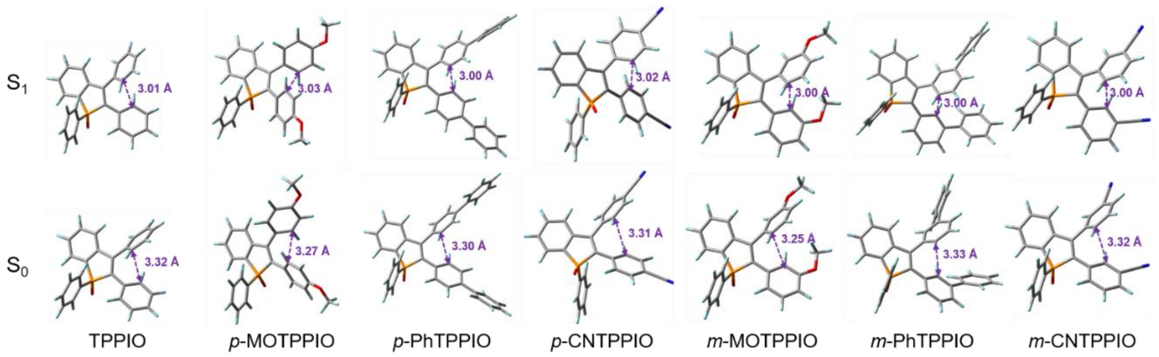


**Figure S20.** Optimized ground-state ($S_0$) and excited-state ($S_1$) geometries of TPPIO derivatives with indicated the smallest C–C distances between two (substitutional) benzenes.

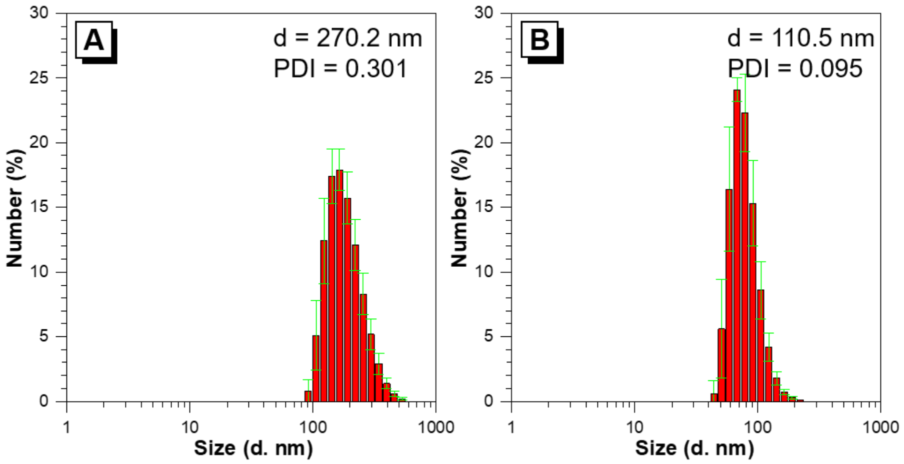


**Figure S21.** Particle size distributions of (A) *p*-MOTPPIO and (B) *p*-MOTBPIO in $H_2O$ with 1 vol% DMSO.

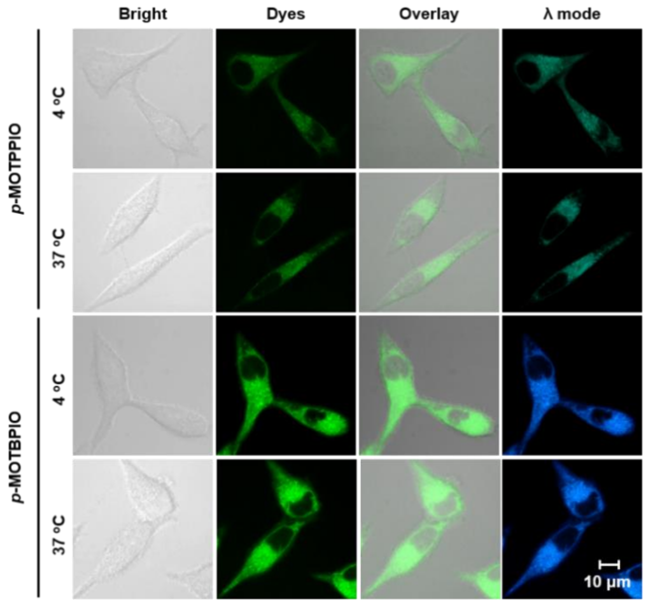


**Figure S22.** CLSM images of HeLa cells after treatment with (A) *p*-MOTPPIO (5 μM) and (B) *p*-MOTBPIO (5μM) for 1 h at different temperatures.

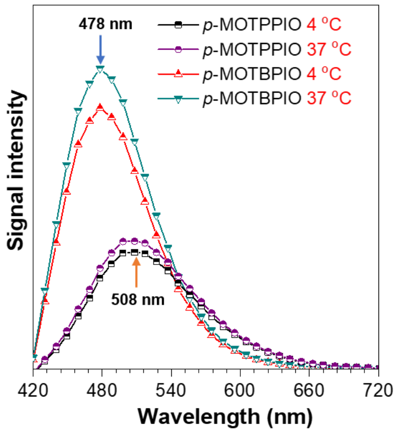


**Figure S23.** Plots of emission signal intensity *vs.* tacking wavelength of *p*-MOTPPIO and *p*-MOTBPIO at different temperatures.

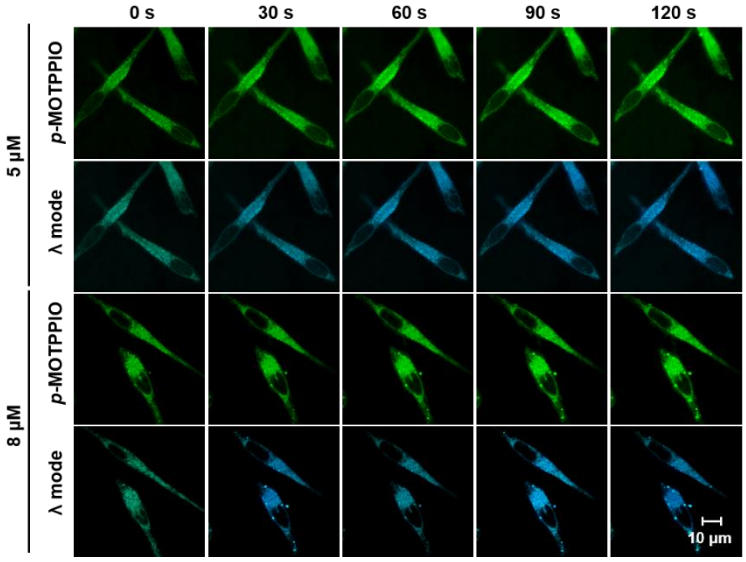


**Figure S24.** CLSM images in channel and lambda (λ) modes of HeLa cells incubated with different concentrations of *p*-MOTPPIO for 1h at continuous irradiation by 405 nm laser.

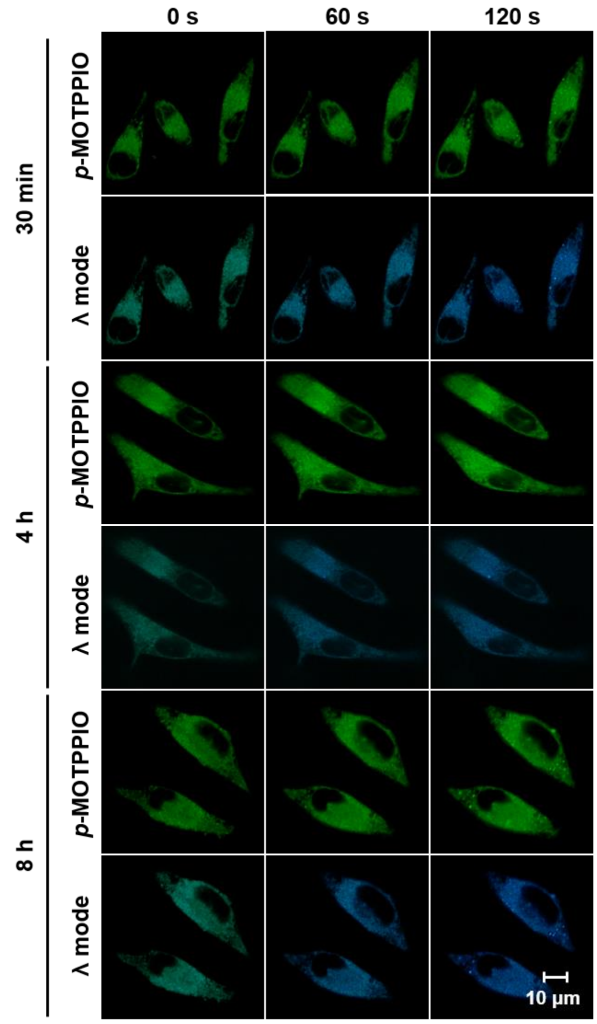


**Figure S25.** CLSM images in channel and lambda (λ) modes of HeLa cells incubated with *p*-MOTPPIO (5 µM) for different time at continuous irradiation by 405 nm laser.

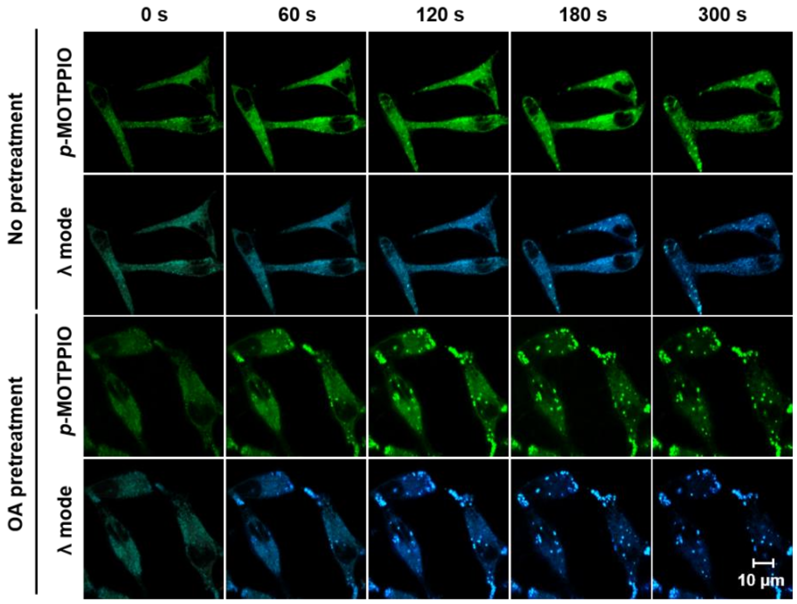


**Figure S26.** CLSM images in channel and lambda (λ) modes of HeLa cells (with or without oleic acid pretreatment) incubated with *p*-MOTPPIO (5 µM) for 1 h at continuous irradiation by 405 nm laser.

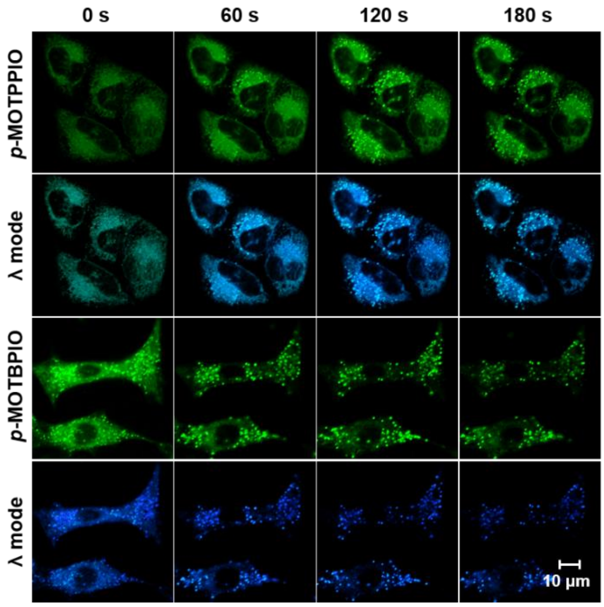


**Figure S27**. CLSM images in channel and lambda (λ) modes of A549 cells (without oleic acid pretreatment) incubated with *p*-MOTPPIO (5 μM) for 1h at continuous irradiation by 405 nm laser.

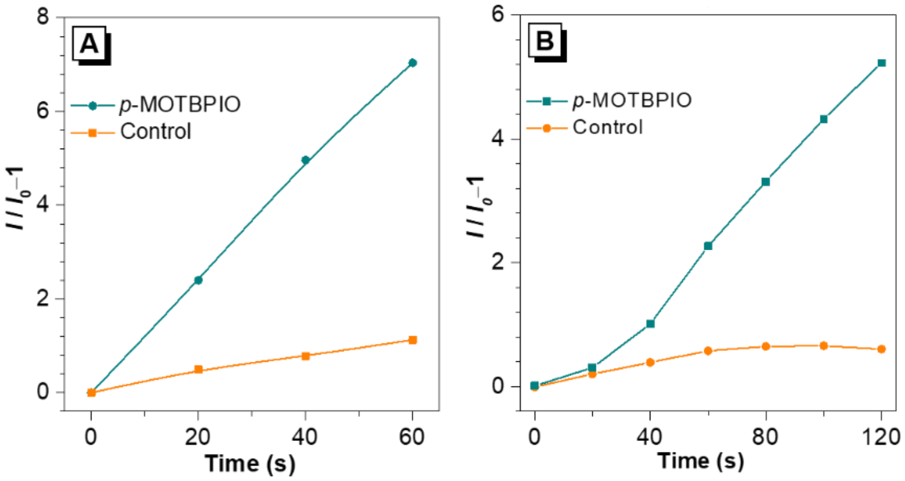


**Figure S28**. The plot of relative signal intensity of (A) DCFH-DA and (B) Liperfluo against irradiation time in *p*-MOTBPIO (5 μM)-treated HeLa cells.

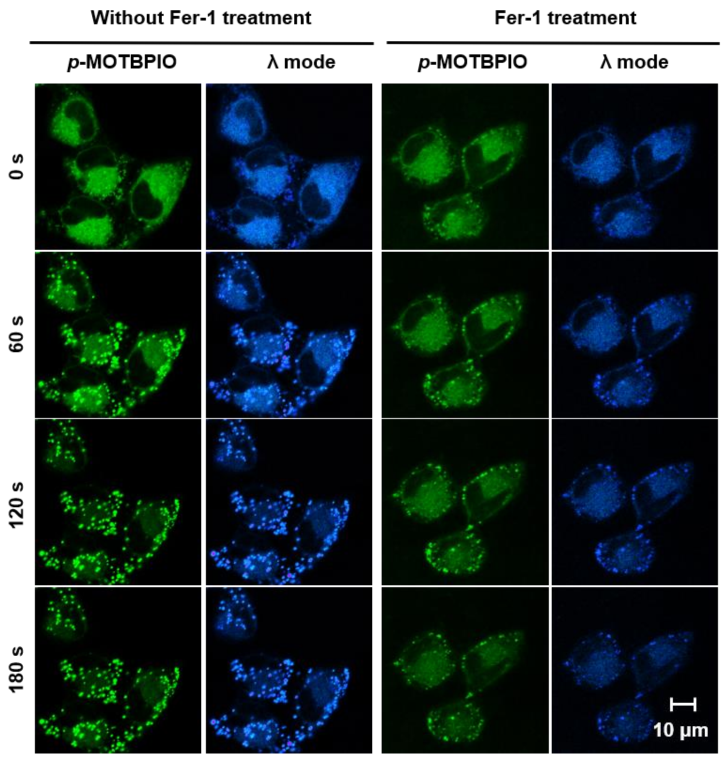


**Figure S29**. CLSM images in channel and lambda (λ) modes of oleic acid-pretreated HeLa cells incubated with *p*-MOTPPIO (5 μM) for 1h and then treated with or without Fer-1 (30 min) at continuous irradiation by 405 nm laser.

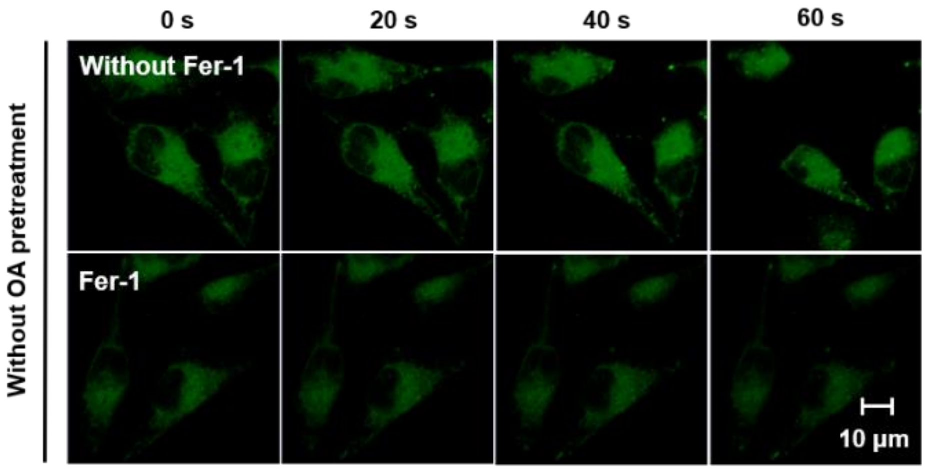


**Figure S30.** CLSM images of HeLa cells (without oleic acid pretreatment) incubated with *p*-MOTBPIO (5 μM) for 1 h and then treated with or without Fer-1 (30 min) at continuous irradiation by 405 nm laser.

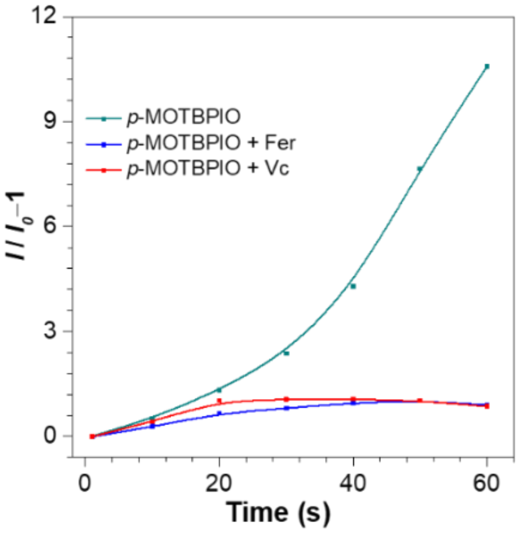


**Figure S31.** The plots of relative signal intensity *vs.* irradiation time in LD area after different treatments.

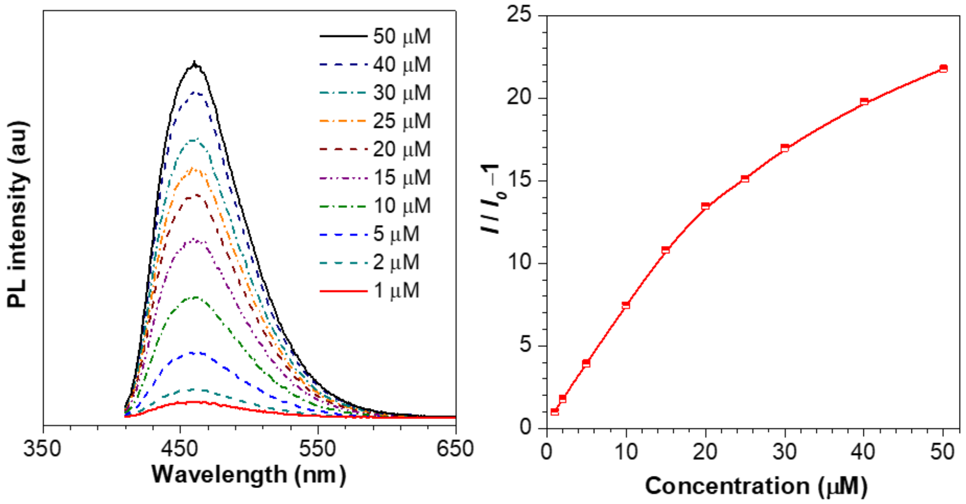


**Figure S32**. PL spectra and plots of $I/I_0 - 1$ *vs.* concentration for *p*-MOTBPIO in 1 mM triglyceride solution (DMSO).

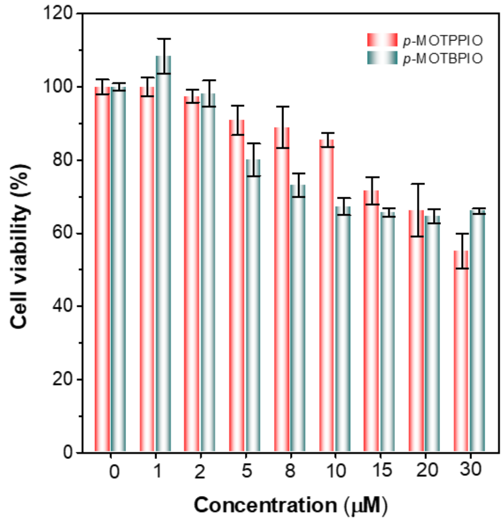


**Figure S33**. Cell viability of L929 cells after treatment with a range of concentrations of *p*-MOTPPIO and *p*-MOTBPIO under dark condition.

**Table S1.** The photoreaction yields of TBPIO derivatives in solutions under $N_2$ and air atmospheres.

| | Yield (%) | |
|---|---|---|
| | $N_2$ | Air |
| TBPIO | 92.0 | 87.5 |
| *p*-MOTBPIO | 72.1 | 32.5 |
| *p*-PhTBPIO | 80.3 | 40.3 |
| *p*-CNTBPIO | 90.5 | 85.6 |
| *m*-MOTBPIO-1 | 1.4 | / |
| *m*-MOTBPIO-2 | 25.4 | / |
| *m*-MOTBPIO-3 | 8.7 | / |
| *m*-MOTBPIO-4 | 35.8 | / |

**Table S2.** The fluorescence quantum yields of TPPIO derivatives in solutions and solid states (films).

| | solution state | solid state | |
|---|---|---|---|
| | $\Phi_F$ [a] (%) | $\Phi_F$ [b] (%) | $\alpha_{AIE}$ |
| *m*-MOTPPIO | 1.2 | 8.1 | 6.75 |
| *m*-PhTPPIO | 2.6 | 38.5 | 14.81 |
| *m*-CNTPPIO | 1.6 | 31.3 | 19.56 |
| *p*-MOTPPIO | 5.5 | 93.3 | 16.96 |
| *p*-PhTPPIO | 12.4 | 75.5 | 6.09 |
| *p*-CNTPPIO | 2.3 | 53.3 | 23.17 |

[a] In toluene solution ($1.0 \times 10^{-5}$ M). [b] Fluorescence quantum yield determined by a calibrated integrating sphere. [c] AIE activity calculated by $\Phi_F$ (solid)/$\Phi_F$ (solution).

**Table S3.** The bond lengths (Å) and dihedral angles (°) of PIO derivates optimized theoretically in the $S_0$ and $S_1$ states.

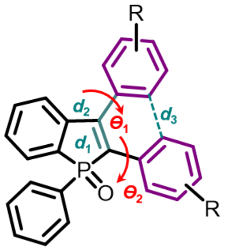


| | $S_0$ | | | | | $S_1$ | | | | |
|---|---|---|---|---|---|---|---|---|---|---|
| | $\Theta_1$ | $\Theta_2$ | $d_1$ | $d_2$ | $d_3$ | $\Theta_1$ | $\Theta_2$ | $d_1$ | $d_2$ | $d_3$ |

| | | | | | | | | | | |
|---|---|---|---|---|---|---|---|---|---|---|
| TPPIO | –53.52 | –38.54 | 1.36 | 1.48 | 3.32 | –34.48 | –15.68 | 1.46 | 1.45 | 3.01 |
| *p*-MOTPPIO | 57.34 | 36.14 | 1.36 | 1.48 | 3.27 | 33.54 | 16.22 | 1.45 | 1.45 | 3.03 |
| *p*-PhTPPIO | –53.02 | –37.72 | 1.36 | 1.48 | 3.30 | –33.26 | –14.18 | 1.45 | 1.44 | 3.00 |
| *p*-CNTPPIO | –54.21 | –39.33 | 1.35 | 1.48 | 3.31 | –34.34 | –15.83 | 1.46 | 1.45 | 3.02 |
| *m*-MOTPPIO | –56.52 | –33.03 | 1.36 | 1.48 | 3.25 | –36.39 | –14.00 | 1.46 | 1.45 | 3.00 |
| *m*-PhTPPIO | –55.15 | –37.99 | 1.35 | 1.48 | 3.33 | –35.33 | –14.54 | 1.46 | 1.45 | 3.00 |
| *m*-CNTPPIO | –53.45 | –41.10 | 1.35 | 1.48 | 3.32 | –34.30 | –14.52 | 1.46 | 1.45 | 3.00 |

**Reference**

[1] Z. Zhuang, F. Bu, W. Luo, H. Peng, S. Chen, R. Hu, A. Qin, Z. Zhao, B. Z. Tang, *J. Mater. Chem. C* **2017**, *5*, 1836–1842.

[2] S. Oshima, H. Ohinata, T. Matsuno, K. Takasawa, Y. Watanabe, K. Fujinage, G. W. Stevens, Y. Komatsu, *Anal. Sci.* **2021**, *37*, 613–617.

[3] O. Berger, A. Kaniti, C. T. van Ba, H. Vial, S. A. Ward, G. A. Biagini, P. G. Bray, P. M. O'Neill, *ChemMedChem* **2011**, *6*, 2094–2108.

[4] J. Wu, B. Qian, Y. Liu, Y. Shang, C*hemistrySelect* **2020**, *5*, 10269–10275.

[5] M. J. Mio, L. C. Kopel, J. B. Braun, T. L. Gadzikwa, K. L.; Hull, R. G. Brisbois, C. J. Markworth, P. A. Grieco, *Org. Lett.* **2002**, *4*, 3199–3202.

[6] T. Lu, F. Chen, *J. Comput. Chem.* **2012**, *33*, 580–592.

[7] W. Humphrey, A. Dalke, K. Schulten. *J. Molec. Graphics* **1996**, *14*, 33–38.
